# Photoacoustic mediated multifunctional tumor antigen trapping nanoparticles inhibit the recurrence and metastasis of ovarian cancer by enhancing tumor immunogenicity

**DOI:** 10.1186/s12951-022-01682-5

**Published:** 2022-11-03

**Authors:** Xiaowen Zhong, Chenyang Li, Guangzong Zhao, Mengmeng Li, Shuning Chen, Yang Cao, Qi Wang, Jiangchuan Sun, Shenyin Zhu, Shufang Chang

**Affiliations:** 1 Department of Obstetrics and Gynecology, The Second Affiliated Hospital of Chongqing Medical University, Chongqing, 400010 People’s Republic of China; 2 Chongqing Key Laboratory of Ultrasound Molecular Imaging, The Second Affiliated Hospital of Chongqing Medical University, Chongqing, 400010 People’s Republic of China; 3 State Key Laboratory of Ultrasound in Medicine and Engineering, Chongqing Medical University, Chongqing, 400016 People’s Republic of China; 4 Department of Pharmacy, The First Affiliated Hospital of Chongqing Medical University, Chongqing, 400042 People’s Republic of China

**Keywords:** Antigen capture, Immunogenic cell death, Tumor immunotherapy, Photodynamic therapy, Photothermal therapy, Photoacoustic imaging

## Abstract

The hypoimmunogenicity of tumors is one of the main bottlenecks of cancer immunotherapy. Enhancing tumor immunogenicity can improve the efficacy of tumor immunotherapy by increasing antigen exposure and presentation, and establishing an inflammatory microenvironment. Here, a multifunctional antigen trapping nanoparticle with indocyanine green (ICG), aluminum hydroxide (Al(OH)_3_) and oxaliplatin (OXA) (PPIAO) has been developed for tumor photoacoustic/ultrasound dual-modality imaging and therapy. The combination of photothermal/photodynamic therapy and chemotherapy induced tumor antigen exposure and release through immunogenic death of tumor cells. A timely capture and storage of antigens by aluminum hydroxide enabled dendritic cells to recognize and present those antigens spatiotemporally. In an ovarian tumor model, the photoacoustic-mediated PPIAO NPs combination therapy achieved a transition from “cold tumor” to “hot tumor” that promoted more CD8^+^ T lymphocytes activation in vivo and intratumoral infiltration, and successfully inhibited the growth of primary and metastatic tumors. An in situ tumor vaccine effect was produced from the treated tumor tissue, assisting mice against the recurrence of tumor cells. This study provided a simple and effective personalized tumor vaccine strategy for better treatment of metastatic and recurrent tumors. The developed multifunctional tumor antigen trapping nanoparticles may be a promising nanoplatform for integrating multimodal imaging monitoring, tumor treatment, and tumor vaccine immunotherapy.

**Supplementary Information:**

The online version contains supplementary material available at 10.1186/s12951-022-01682-5.

## Background

Ovarian cancer is one of the representatives of metastatic and recurrent tumors [1]. The mortality rate of ovarian cancer ranks first among gynecological malignancies due to the difficulty of early diagnosis [2]. Small residual lesions after cytoreductive surgery are important reasons for its recurrence. The metastasis of intraperitoneal dissemination of ovarian cancer poses a significant challenge to traditional methods to remove the residual disease [3, 4]. Therefore, it is imperative to find new diagnostic and therapeutic strategies that can effectively eradicate both in situ tumors and metastatic lesions of ovarian cancer.

Cancer immunotherapy is a vital strategy to inhibit tumor recurrence and metastasis by activating the autoimmune system to eliminate heterogeneous tumor cells. Nonspecific immunotherapy methods, including cytokine therapy [5], immune checkpoint blockade therapy [6], and adoptive T cell metastasis [7], have been affirmed in the treatment and prevention of cancer metastasis and recurrence. However, these methods are limited by individual response variability, low treatment responsiveness, and immunotoxicity. Specific immunotherapy based on tumor-specific antigens (TSAs) or tumor-associated antigens (TAAs) is an ideal strategy for tumor immunotherapy [8]. Current sources of TSAs include genes expressing tumor antigens, tumor-related proteins or polypeptides, inactivated autologous or allogeneic tumor tissue components, autologous or allogeneic tumor tissue, or cell-derived complexes [9]. Tumor vaccines based on autologous or allogeneic tumor tissue are prepared by invasively obtaining tumor tissue, which is extremely complicated. At the same time, its efficacy is constrained by tumor heterogeneity and blurred immune focus [10, 11]. It is also not practical to prepare a personalized tumor vaccine for individual tumor tissues. Tumor inactivation in situ can theoretically solve the above problems. A personalized in situ vaccine can be generated by activating the immunogenicity of a deadly tumor. However, whether in situ inactivated tumor tissue can produce tumor vaccine effects depends on the exposure abundance of tumor antigens, the processing and presentation of tumor antigens by antigen presenting cells (APCs), the activation and proliferation of lymphocytes, intratumoral infiltration of cytotoxic T lymphocytes (CTLs). Insufficient exposure and presentation of tumor antigens are the leading causes of hypoimmunogenicity and lack of CTL infiltration in tumor tissue [12]. Studies have shown that tumor immunotherapy needs to overcome the two bottlenecks of insufficient infiltration of CTLs in tumor tissues and an immunosuppressive tumor microenvironment to exert reliable efficacy [13]. Its essence is the lack of tumor immunogenicity and the existence of immune tolerance. Transforming a low immunogenic “cold tumor” into a high immunogenic “hot tumor” is key to solving the scientific problem of tumor in situ vaccines.

Immunogenic cell death (ICD) is a distinct form of apoptosis that occurs in tumor cells after the action of certain chemotherapeutic drugs, such as anthracyclines, photothermal therapy (PTT), and photodynamic therapy (PDT) [14]. ICD is characterized by the release of molecular distress signals with dangerously associated molecular patterns, including adenosine triphosphate (ATP), high mobility group 1 (HMGB1) proteins, calreticulin (CRT), and heat shock proteins (HSP) 70 and 90 [15]. ICD tumor cells release many TAAs to promote the immune cycle of tumors, which is an effective way to improve antigen exposure.

With the popularization of cancer nanomedicine and nanomedicine, the integration of tumor diagnosis and treatment has made a breakthrough [16, 17]. Combination therapy often results in better outcomes than monotherapy. Liquid fluorocarbon-photoacoustic nano contrast agent mediated PTT and PDT have unique advantages in tumor therapy. On the one hand, the multifunctional liquid fluorocarbon-photoacoustic nanocontrast agent can realize photoacoustic (PA) imaging through the “photovaporization droplet (optical droplet vaporization, ODV) effect”, which perfectly combines the high selectivity of optical imaging and the deep penetration advantages of ultrasound imaging [18, 19]. On the other hand, PTT/PSDT mediated multifunctional nano contrast agent can induce tumor cell death, promote tumor antigen exposure, recruit inflammatory cell aggregation, promote the release of inflammatory factors, and form tumor inflammatory microenvironment through ultrasound cavitation, reactive oxygen species (ROS) and chemical killing mechanism [20]. Our previous study has shown that liquid fluorocarbon contrast agent encapsulated OXA and ICG (OIX-NPs) can not only promote ovarian cancer cell apoptosis, but also effectively induce TAAs exposure [21]. However, OIX-NPs mediated combination therapy can only inhibit primary tumor growth but cannot resist the secondary attack of tumor cells, which may be related to the lack of recognition and effective presentation of tumor antigens by APCs [22]. Studies have shown that capturing antigens released by tumor cells can promote the recognition and extraction of antigens by DC and induce a more powerful specific antitumor immune response [23, 24].

Aluminum hydroxide is the first human adjuvant approved by the Food and Drug Administration (FDA). Aluminum-based adjuvants (ABAs) are currently the safest and most widely used immune adjuvants globally. Aluminum hydroxide captures different antigens through electrostatic adsorption, ligand exchange, and hydrophobic action [25]. Soluble antigens become granular antigens by binding to ABAs. Captured antigens can be highly aggregated on and within aluminum adjuvants without chemical property changes [26, 27]. Tumor antigens captured by aluminum hydroxide form an antigen reservoir, which provides temporal and spatial guarantees for the aggregation of APCs and CTLs. Aluminum-containing adjuvants enhance immune responses through dendritic cell (DC) stimulation, complement activation, and chemokine release induction [28]. Nanoscale aluminum hydroxide can more efficiently promote the interaction between antigens and APCs [29, 30].

**Fig. 1:**
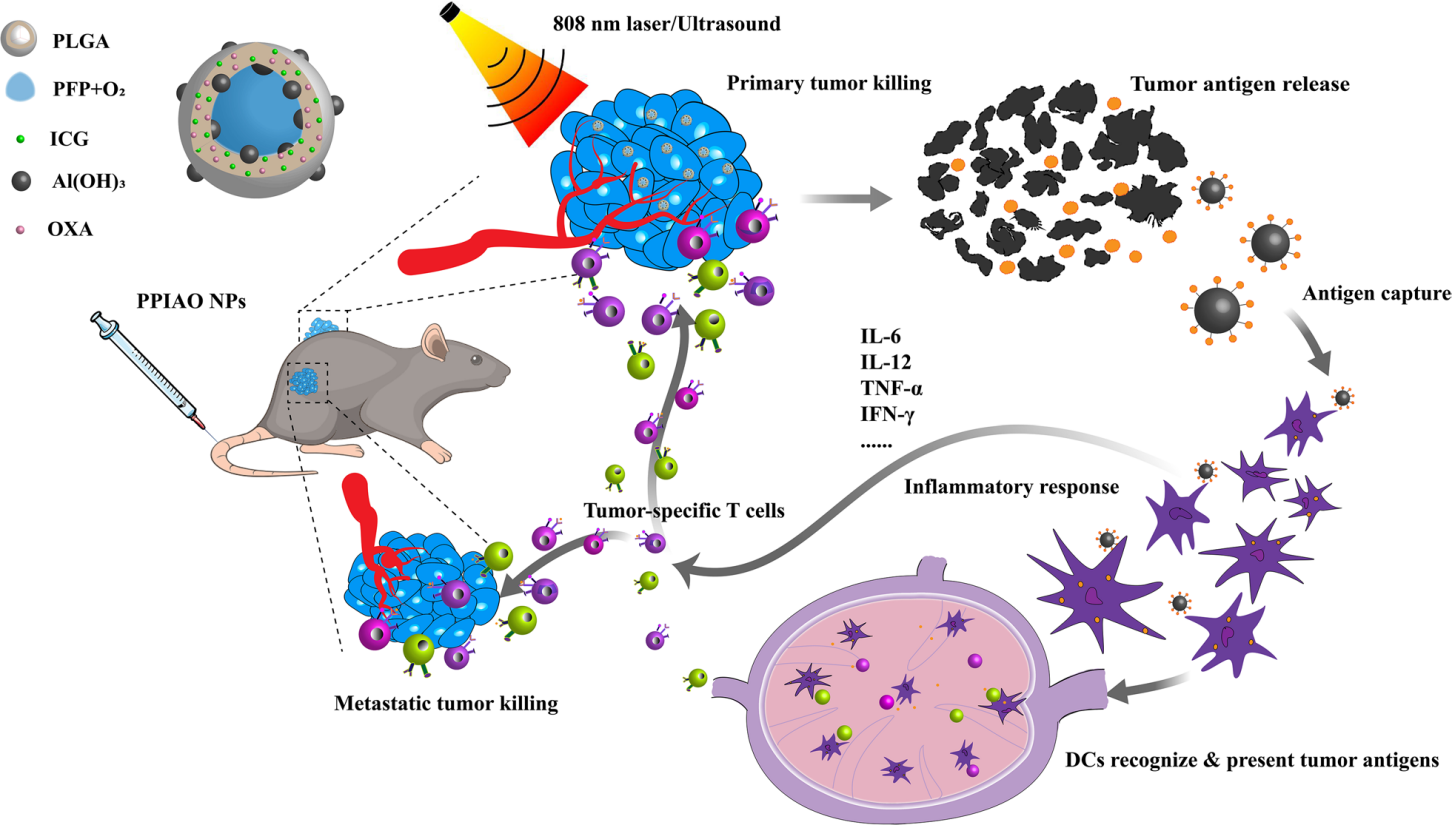
Schematic diagram of PPIAO NPs mediated photothermal/photodynamic therapy improving tumor immunotherapy

We constructed multifunctional tumor antigen trapping nanoparticles (PPIAO NPs) integrating dual-modal imaging and chemotherapy/PTT/PDT/immunotherapy through a nano drug delivery system. Efficacy of combination therapy evaluated in C57BL/6 mouse ovarian cancer model. In this study, in addition to the typical enhanced permeability and retention (EPR) effect of nanoparticles, local hyperthermia combined with ultrasound-targeted microbubble destruction technology overcame the tumor vascular endothelial barrier and improved the penetration and aggregation of nanoparticles in the tumor. In vivo, treatment of the primary tumor promoted TAAs exposure. Tumor antigens captured and stored by nano aluminum hydroxide effectively promoted DC maturation and antigen presentation, which induced more activation and intratumoral infiltration of CTLs. Then, the metastatic and residual tumor cells were surrounded by activated specific antitumor immune killers. Tumor recurrence was prevented by antitumor immune memory (Fig. 1). This study provides a simple and effective personalized tumor vaccine strategy for better treatment of metastatic and recurrent tumors.

## Materials and methods

### Materials

PEGylated poly (lactic-*co*-glycolic acid, lactide:glycolide = 50:50, PLGA 20,000 Da MW, PEG 2000 Da MW) (PLGA-PEG2000) was obtained from Ruixi Biotechnology (Xian, China). Polyvinyl alcohol (PVA) and ICG were purchased from Sigma Aldrich (St. Louis, MO, USA). Perfluoropentane (PFP) was purchased from Strem Chemicals (MA, USA). OXA was purchased from MedChemExpress (NJ, USA). Nano aluminum hydroxide (Al(OH)_3_) was purchased from Ruixi Biotechnology (Xian, China). 4′,6-Diamidino-2-phenylindole (DAPI), ATP Assay Kit, 1,1′-dioctadecyl-3,3,3′,3′-tetramethylindocarbocyanine perchlorate (DiI), Hoechst 33342 Live Cell Stain, 2′,7′-dichlorofluorescein diacetate (DCFH-DA), LDH Cytotoxicity Assay Kit were obtained from Beyotime Biotechnology (Chongqing, China). Bradford Assay Kit was obtained from Abcam (Cambridge, UK). Calcein/Propidium Iodide (CAM/PI) was purchased from Santa Cruz Biotechnology (TX, USA). OVA-FITC, bovine serum albumin-HRP (BSA-HRP), and ACK lysis buffer were obtained from Soleibo Technology (Beijing, China). Annexin V-FITC/PI was purchased from Elabscience (Wuhan, China). Singlet oxygen sensor green (SOSG) fluorescent probe was obtained from Invitrogen (NY, USA). A CFSE Cell Division Tracker Kit was purchased from BioLegend (CA, USA). All chemicals were analytical grade. All antibody information for flow cytometry (FCM) and immunofluorescence is annotated in Additional file 1.

### Cell culture and animal model

Mouse epithelial ovarian cancer ID8 cells were provided by Dr. Katherine Roby (University of Kansas Medical Center, USA). All cells were maintained in a cell incubator (37 °C, 5% CO_2_) and used for the experiment when they reached 80% confluence. Dulbecco’s modification of Eagle’s medium Dulbecco (DMEM) containing 10% fetal bovine serum (FBS), 50 µg/L penicillin, and 50 µg/L streptomycin was used for cell culture. Female C57BL/6 mice (4–6 weeks old, 18–22 g) were purchased from the Laboratory Animal Center of Chongqing Medical University (Chongqing, China) and treated under the Guidelines for the Care and Use of Laboratory Animals. All animal experiments were approved by the Animal Ethics Committee of Chongqing Medical University. ID8 cells (100 µL, 1 × 10^6^ cells/mL) were injected subcutaneously into the right-back flank of mice to establish a solid tumor. Mice received treatment when the tumor volume reached 200 mm^3^. The tumor volumes were calculated as 0.5 × length × width^2^.

### Preparation of PPIAO NPs

PPIAO NPs were prepared by a modified multistep emulsion method [21, 22, 31]. (i) 1 mL of ICG aqueous solution (1.5 mg/mL), 1 mL of OXA aqueous solution (3 mg/mL), and 1 mL of Al(OH)_3_ aqueous solution (2 mg/mL) were fully emulsified with 200 µL of oxygen-carrying PFP for 60 s. The first emulsification step promoted the complete dissolution of the drug and the uniform dispersion of Al(OH)_3_. (ii) 2 mL of PLGA-PEG2000 dichloromethane solution (25 mg/mL) was added to the above solution, and sonicated for 3 min (time on 5 s, time off 5 s, power 25%). (iii) 2 mL of PVA solution (5% w/v) was added to the second step solution, and sonicated for 3 min. (iv) 10 mL of isopropanol solution (2% w/v) was added to solidify the nanoparticle shell. The above milky solution was magnetically stirred under low-temperature protection for 12 h to fully remove the organic solvent. Finally, the milky solution was collected and centrifuged (12,000 rpm, 4 °C, 10 min) to remove the cloudy supernatant. PPIO NPs (without Al(OH)_3_) were synthesized by a similar method. DiI was added to dichloromethane to synthesize DiI-labeled nanoparticles (DiI-NPs).

### Characterization of PPIAO NPs

The morphology and structure of PPIAO NPs were characterized by transmission electron microscope (TEM, Hitachi H-7600, Hitachi Ltd., Tokyo, Japan) and scanning electron microscopy (SEM, AZtecLive Ultim Max 100, Oxford Instruments). The average particle size and zeta potential of nanoparticles were measured by a dynamic laser light scattering system (DLS, Malvern Instruments, Malvern, UK). To evaluate the stability of nanoparticles, the average particle size of nanoparticles dispersed in DMEM, 10% BSA, double distilled water (ddH_2_O), and phosphate buffered saline (PBS) was measured on days 0, 7, 14, 21, and 28 after synthesis. A UV-VIS-NIR spectrophotometer (UV-3600, Shimadzu, Japan) was used to quantitatively assess the loaded ICG content. Fluorescence spectra of nanoparticles were measured by spectrofluorophotometer (Cary Eclipse, Agilent Technologies) with excitation at 760 nm and recorded from 760 to 860 nm. Encapsulated aluminum hydroxide was determined by Fourier transform infrared spectrometer (FTIR, Nicolet iS50, Thermo Fisher Scientific), X-ray photoelectron spectroscopy (XPS, Thermo Scientific K-Alpha+), and energy dispersive spectroscopy (EDS, AZtecLive Ultim Max 100).

### Photothermal conversion and photoacoustic dynamics performance

Temperature and infrared thermal images of PPIAO NPs aqueous suspensions (ICG 35.67 µg/mL, OXA 15.21 µg/mL) under irradiation of different intensities (0.5, 1.0, 1.5, 2.0, 2.5 W/cm^2^) of an 808 nm laser (Zhongchuan Optoelectronics Technology, Xian, China) were recorded with an infrared thermal imager (FORTRICE 226, China). The photoacoustic dynamic effects of PPIAO NPs were evaluated by SOSG. As previously described [32], 10 µL of SOSG (500 µM) was added to 2 mL of the sample solution (PPIAO NPs, PPIO NPs, and free ICG with an ICG concentration of 35.67 µg/mL). The above solution was irradiated with an 808 nm laser (2.0 W/cm^2^, 5 min) and ultrasound (1 W/cm^2^, 5 min) (Chongqing Key Laboratory of Ultrasound Molecular Imaging, Chongqing, China). The fluorescence intensity curves of each group were detected by spectrofluorophotometer (λ_excitation_/λ_emission_ = 504 nm/525 nm).

### Laser/ultrasound responsive OXA release

The content of OXA in the nanoparticles and the laser/ultrasound responsive drug release efficiency were detected by high-performance liquid chromatography (HPLC, Agilent 1260 Infinity II, Agilent Technologies). Briefly, the aqueous nanoparticles were irradiated with 808 nm laser and ultrasound (808 nm laser 2.0 W/cm^2^ × 5 min and ultrasound 1 W/cm^2^ × 5 min, OXA 15.21 µg/mL). After centrifugation, the supernatant was collected, and the concentration of OXA in the supernatant was detected.

### PA/ultrasound dual-modality imaging

A 3% (w/v) agarose gel model was used for in vitro dual-mode imaging observation. According to previous reports [21, 22, 31], standard B-mode and contrast-enhanced ultrasound (CEUS) modes of PBS, free ICG, PPIAO NPs, and PPIO NPs suspensions (ICG concentrations of 35.67 µg/mL) before and after 808 nm laser and ultrasound were observed by the linear probe of a diagnostic ultrasound machine (5–12 MHz) (MyLab 90, Esaote, Italy). Image analysis software (Model: DFY) was used to analyze the echo intensity (EI). The PA performance of PPIAO NPs was evaluated by the Vevo LAZR Photoacoustic Imaging System (VisualSonics Inc., Toronto, Canada). PA imaging was performed with a laser with an excitation wavelength of 780 nm. The PA value of each group was quantified by Vevo LAZR software. In vivo dual-modality imaging of PPIAO NPs was assessed in a C57BL/6 mouse ID8 ovarian cancer subcutaneous transplant model (n = 3). Ultrasound and PA images were collected at different times (Pre, 2, 4, 6, 12, 24 h) after intravenous injection of PPIAO NPs (ICG 7.36 mg/kg, OXA 3 mg/kg).

### Biosafety, pharmacokinetics (pK) and biodistribution

The IC_50_ of free OXA and the cytotoxicity of nanoparticles were detected by the CCK-8 method and the IC_50_ of PPIAO + L.U. group (808 nm laser 2.0 W/cm^2^ × 5 min and ultrasound 1 W/cm^2^ × 5 min). The biosafety of PPIAO NPs was assessed in healthy female C57BL/6 mice. Twenty one mice were randomly divided into 7 groups (n = 3), including the normal saline group (intravenous injection of normal saline), 1, 7, 14, 21, and 28-day group (days after intravenous injection of PPIAO NPs). The blood and major organs (heart, liver, spleen, lung, kidney) were collected. Routine blood tests and serum biochemistry (alanine aminotransferase, aspartate aminotransferase, total bilirubin, creatinine, blood urea nitrogen, creatine kinase, and l-lactate dehydrogenase) were performed. The tissue structure changes in the major organs were analyzed by HE staining. In addition, the brain cell morphology and structure in the mouse brain tissue were observed by HE staining 90 days after administration. The pharmacokinetics (pK) and biodistribution of PPIAO NPs were evaluated in a C57BL/6 mouse ID8 ovarian subcutaneous transplant model. Blood, major organs and tumors were collected at different times (0.5, 1, 2, 4, 6, 8, 12, 24, 48 h) after intravenous injection of PPIAO NPs solution (OXA 3 mg/kg). The Pt concentration was analyzed by inductively coupled plasma-mass spectrometry (ICP-MS, NexION 300X, PerkinElmer).

### In vitro cellular uptake

ID8 tumor cells (1 × 10^5^ cells) were grown in confocal dishes. After the cells adhered, DiI-labeled PPIAO NPs (DiI-NPs) (OXA 15.21 µg/mL) were added and coincubated for different times (0.5, 1.0, 1.5, 2 h). Then, tumor cells were fixed with 4% paraformaldehyde (PFA) and nuclei were labeled with DAPI. Nanoparticle uptake by tumor cells was observed by confocal laser scanning microscope (CLSM, Nikon A1, Japan). ID8 cells grown in six-well plates were incubated with DiI-NPs and harvested by trypsinization. Flow cytometry (FCM, Becton–Dickinson, Franklin Lakes, NJ) was used to measure the phagocytosis rate of nanoparticles. Tumor bodies were formed by ID8 cells cultured in low-adsorption well plates for 7 days and cocultured with DiI-labeled PPIAO NPs. Nuclei were labeled with Hoechst 33342 Live Cell stain. Then, the infiltration and aggregation of nanoparticles in tumor bodies were observed by 3D imaging with CLSM.

### In vitro antitumor efficacy

Croups in cell experiments included the control group, ICG + L.U.group, free OXA group, PPIO + L.U. group, PPIAO + L.U. group, and PPIO + Al + L.U. group (ICG 35.67 µg/mL, OXA 15.21 µg/mL). ID8 tumor cells (1 × 10^5^ cells) were grown in confocal dishes. After the cells adhered, the original culture medium was replaced with a fresh medium containing PBS, ICG, free OXA, PPIO NPs, PPIAO NPs, or PPIO NPs + Al(OH)_3_. The cells of the L.U. groups were treated with 808 nm laser and ultrasound after coincubation for 2 h. The generation of intracellular ROS in each group was detected by DCFH-DA. ID8 cells of the same treatment were identified as live/dead by CAM/PI double staining. The apoptosis ratio of the cells was detected by FCM, or the cell viability was measured by CCK-8.

### In vitro antigen exposure

Tumor antigen exposure was detected by immunofluorescence. ID8 cells grown in confocal dishes were treated and placed on ice. Cells were fixed with 4% PFA for 20 min after washing with PBS. The membrane was broken through 0.5% Triton-100 for 10 min. The residual PBS was removed with absorbent paper, blocking solution (10% FBS) was added, and the membrane was incubated for 30 min. The primary antibody (anti-Calreticulin antibody or anti-HMGB1 antibody) was incubated with the samples for 12 h at 4 °C. After washing with PBS, samples were incubated with fluorescent (Alexa Fluor^®^ 488 or Cy5)-labeled goat anti-rabbit IgG H&L (2 µg/mL) for 1 h. Nuclei were labeled by DAPI staining for 10 min. Finally, an anti-fluorescence quencher was added, and the membrane inversion of CRT and the secretion of HMGB1 in ID8 cells were observed by CLSM. In addition, antigen-exposed tumor cells were collected for further detection by FCM. ATP release from ID8 cells in the supernatant was detected by an ATP Assay Kit.

### Antigen capture

First, the protein content captured by PPIAO NPs and nano aluminum hydroxide was estimated by the Bradford method using BSA as a standard. The protein adsorbed by nanoparticles was the amount of total protein minus the amount of protein in the supernatant. The hydrodynamic particle size and zeta potential changes of PPIAO NPs before and after protein adsorption were measured by DLS. Morphological changes before and after aluminum hydroxide capture protein were observed by TEM. The differences between lysed tumor cell proteins and captured proteins were detected by sodium dodecyl sulfate-polyacrylamide gel electrophoresis (SDS-PAGE). Briefly, ID8 cells were cultured with DMEM (FBS free) containing ICG (35.67 µg/mL) and OXA (15.21 µg/mL) for 2 h. After treating the cells with an 808 nm laser and ultrasound, the supernatant was collected and centrifuged (200*g*, 5 min) to remove any insoluble cellular debris. Aluminum hydroxide (5.38 µg/mL) was incubated with the supernatant for 4 h and resuspended in PBS for further detection after washing 3 times with PBS. As reported [23], mass spectrometry analysis was performed by an LC/MS system (Q Exactive™, Thermo). The mass spectral data generated by QE were searched by Protein Discover (V2.2), and the database search algorithm used was Percolator. The database used for the search was the Proteome Reference Database for Mouse in Universal Protein (Universal Protein mouse 20190908. fasta). Combining the known antigens [14, 33–37] with a list of captured proteins obtained from mass spectrometry data, the captured tumor antigens are presented according to the number of containing specific peptides. Further, antigenic peptide sequences and information are available in the tumor antigen database TANTIGEN 2.0 (https://projects.met-hilab.org/tadb).

### DC stimulation in vitro

ID8 (upper layer) and DCs (lower layer) were cocultured in transwell chambers. After the tumor cells in the upper layer were irradiated, the expression of the costimulatory molecules CD11c/CD80/CD86 in DCs in each group was detected by FCM. The content of IL-12 in the supernatant of the chamber was detected by enzyme-linked immunosorbent assay (ELISA). Experiments were carried out according to the operating instructions, and 5 replicate wells were set in each group. DCs were labeled with CFSE Cell Division Tracker, and ID8 cells were stained with DiI. The morphological changes of DCs were observed by CLSM. Furthermore, to observe the internalization of captured antigens by DCs and macrophages, OVA-FITC was captured by DiI-NPs and then incubated with DAPI-labeled DCs and macrophages for 2 h. Antigen uptake by the two APCs was observed by CLSM after washing with PBS.

### In vivo antitumor effect

All in vivo studies were performed in female tumor-bearing C57BL/6 mice. Groups included the control group, ICG + L.U.group, free OXA group, PPIO + L.U. group, PPIAO + L.U. group, PPIO + Al + L.U. group (ICG 7.36 mg/kg, OXA 3.0 mg/kg). Mice in the control group received only PBS. Mice in the free OXA group received only free OXA intravenously. Mice in the ICG + L.U.group, PPIO + L.U. group, PPIAO + L.U. group, and PPIO + Al + L.U. group received the combined treatment of 808 nm laser and ultrasound at the tumor site 6 h after intravenous administration. All treatments were repeated three times on day 1, day 4 and day 7. Sodium pentobarbital was used for mouse anesthesia (35 mg/kg). The temperature change during the treatment was monitored by a thermal infrared imager. The body weight and tumor volume of the mice were recorded every 2 days for 21 consecutive days. On days 8, 11, and 14, blood, tumor, and major organs were collected from mice. Tumor tissues were stained with HE, PCNA and TUNEL. The remaining mice were used for 90-day survival observation. Mice were sacrificed when the tumor volume reached 1500 mm^3^.

### In vivo antigen exposure

The exposed tumor antigens were detected by the immunofluorescence homologous double-labeling method on paraffin sections. Briefly, 4% PFA-fixed paraffin-embedded tumor tissue was sectioned (4 μm). Paraffin sections were dewaxed with alcohol and xylene and hydrated with distilled water. Samples were placed in EDTA antigen retrieval buffer for antigen retrieval at 98 °C for 20 min. Blocking of endogenous peroxidase in the tissue was achieved by incubation with 3% hydrogen peroxide. After washing with PBS, serum was added to the block for 30 min. Tissue sections were first incubated with primary antibody (anti-HMGB1 antibody) for 12 h at 4 °C. HRP-labeled secondary antibody was added and incubated for 1 h at room temperature. Cy5-TSA was added and incubated in the dark for 10 min. After washing 3 times on a destaining shaker, the sections were placed in antigen retrieval buffer and heated in a microwave oven to remove the antibody-TSA complex. Then, sections were incubated with primary antibody (anti-calreticulin antibody) for 12 h at 4 °C. The corresponding Alexa Fluor^®^ 488-labeled secondary antibody was added and incubated for 1 h. DAPI stained nuclei for 10 min. The autofluorescence quencher was incubated for 5 min. Finally, sections were sealed in an anti-fluorescence quencher and scanned by Pannoramic P-MIDI (3D HISTECH, Hungary). Images were analyzed using SlideViewer image analysis software. The efficacy data were representative of three independent experiments.

### Antitumor immune activation

FCM was used to measure the DC maturation and the abundance of T lymphocytes in the spleen and tumor tissues of the mice collected after the above treatment. A single-cell suspension was prepared by enzymatic lysis solution (1% hyaluronidase, 1% collagenase, 0.5% deoxyribose). Red blood cells from spleen tissue were eliminated by ACK lysis buffer. The cells were detected by Live/Dead Cell Staining Kit. Samples were incubated with FC Block (anti-mouse CD16/32 monoclonal antibody) for 5 min on ice before cell staining. CD4^+^ T and CD8^+^ T lymphocytes were labeled with APC anti-mouse CD3 antibody, FITC anti-mouse CD4 antibody, and PC5.5 anti-mouse CD8a. DCs were labeled with FITC anti-mouse CD11c antibody, PE anti-mouse CD86 antibody, and APC anti-mouse CD80 antibody. Analyses were performed using FlowJo V10 software. According to the detection instructions, the secretion levels of IL-6, IL-12, TNF-α, and IFN-γ in the serum of mice after 7 days of treatment were detected by ELISA.

### Abscopal effect

On day-14 and day-10, ID8 cells (100 µL, 1 × 10^6^ cells/mL) were injected subcutaneously into the right-back flank (primary tumor) and left-back flank (distant tumor) of mice. Grouping and treatment parameters were as previously described. On days 1, 4, and 7, the mice were treated with primary tumors. Sodium pentobarbital (1%) was used for mouse anesthesia (35 mg/kg). The temperature change at the tumor site during irradiation was monitored by a thermal infrared imager. The body weight and bilateral tumor volume were recorded every 2 days for 21 days. The infiltration of T lymphocytes in primary tumors and metastases of mice in each group was detected by immunofluorescence 7 days after the last treatment. Similar to the in vivo antigen exposure detection method, intratumoral T lymphocytes were detected by the immunofluorescence homologous double-labeling method on paraffin sections. The antibodies included anti-CD4 antibody, anti-CD8 antibody and the corresponding fluorescently labeled secondary antibody. Sections were stored in an anti-fluorescence quencher. The samples were scanned by Pannoramic P-MIDI. Images were analyzed using SlideViewer image analysis software. Similarly, the abundance of CD8^+^ T lymphocytes in the spleen of mice in each group was detected by FCM using flow cytometry antibodies, including APC anti-mouse CD3 antibody, FITC anti-mouse CD4 antibody, and PC5.5 anti-mouse CD8a. To evaluate the systemic efficacy based on PPIAO + L.U. combination therapy, the in vitro killing activity of mouse spleen lymphocytes against ID8 cells was examined. Cells were counted. A 50:1 ratio of spleen lymphocytes and ID8 cells was coincubated in a cell incubator for 4 h. ID8 cell viability was evaluated by a lactate dehydrogenase detection kit. The level of IFN-γ cytokine secretion in the supernatant was detected by ELISA.

### Vaccine effects and tumor challenges

Tumor bodies were formed by culturing an equal amount of ID8 cells in low-adsorption well plates for 7 days. The original medium was replaced with serum-free medium containing PPIO NPs, PPIAO NPs, or PPIO NPs + Al(OH)_3_. After coincubation for 2 h, 808 nm laser and ultrasound were performed in 24-well plates. The tumor bodies and supernatants were collected and inoculated into the root of the left hind leg of C57BL/6 mice (4 weeks old). Groups included the control (PBS) group, PPIO + L.U. group, PPIAO + L.U. group and PPIO + Al + L.U. group. Each group of mice was vaccinated twice with tumor cell vaccines and separated by 7 days. One week after the last vaccination, all mice were subcutaneously inoculated with the same batch of ID8 tumor cells (1 × 10^6^ cells/mL). The tumor growth of all mice was observed. Three mice were randomly selected from each group to collect the spleen 7 days after the inoculation of the cell vaccine. The abundance of memory T lymphocytes (CD3^+^ /CD8^+^ /CD44^+^) was detected by FCM with APC anti-mouse CD3 antibody, PC5.5 anti-mouse CD8a antibody, and ER780 anti-mouse CD44 antibody. Tumor cell challenge experiments were performed on unilateral tumor-bearing mice in the PPIAO + L.U. group that received three combined treatments (n = 5). Seven days after the last treatment, an equal amount (1 × 10^6^ cells/mL) of ID8 cells was inoculated on the left-back of all mice. Subcutaneous tumor growth in mice was then observed.

### Statistical analysis

Data were presented as the mean ± SD. Significance was determined by one-way ANOVA with Bonferroni post-test or Dunnett post-test using GraphPad Prism 7.0. A value of *p* < 0.05 was considered statistically significant. *Compared with the control group, *p < 0.05, **p < 0.01, ***p < 0.001, ****p < 0.0001; ^#^was the comparison between groups, ^#^*p* < 0.05, ^##^*p* < 0.01, ^###^*p* < 0.001, ^####^*p* < 0.0001; ns, *p* > 0.05.

## Results and discussion

### Characterization of PPIAO NPs

**Fig. 2:**
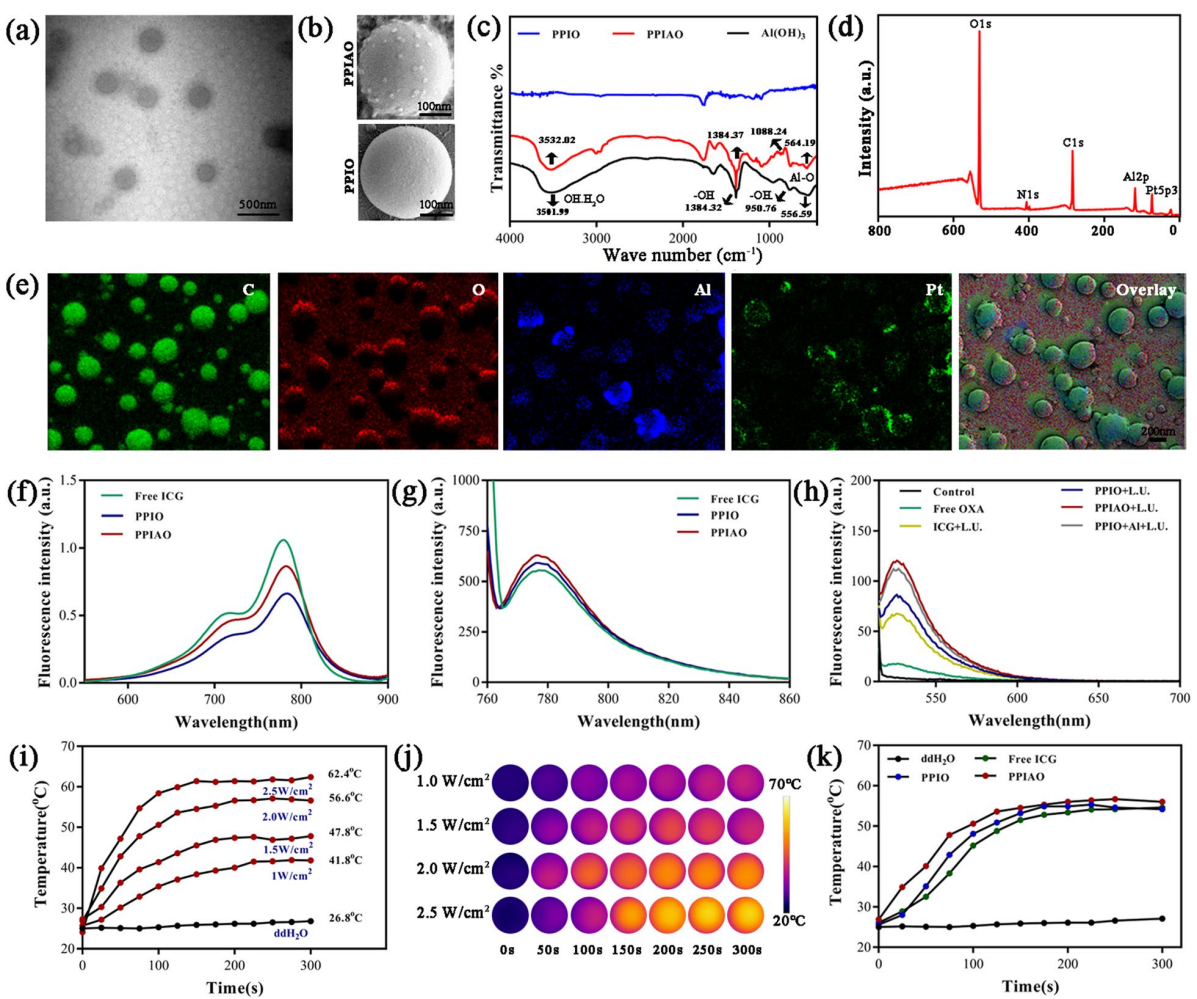
Characterization of PPIAO NPs. **a** Transmission electron microscopy image of PPIAO. Scale bar 500 nm. **b** Scan electron microscopy image of PPIAO and PPIO. Scale bar 100 nm. **c** FTIR spectrum of PPIAO, PPIO and Al(OH)_3_. **d** X-ray photoelectron spectroscopy spectrum of PPIAO. **e** Energy dispersive spectroscopy analysis of PPIAO. The corresponding elemental mapping images. Scale bar 200 nm. **f** UV–Vis absorbance spectra and **g** Fluorescence spectra of PPIAO, PPIO and free ICG. Evaluation of the optical properties of ICG in nanoparticles. **h** SOSG detection of ^1^O_2_ generation. λ_excitation_/λ_emission_ = 504 nm/525 nm. **i**,** j** Thermal infrared images of PPIAO aqueous suspensions irradiated with different intensities of the 808 nm laser. **k** Temperature variation of PPIAO, PPIO and free ICG, compared with ddH_2_O.

PPIAO NPs were spherical with granular structures on the surface under TEM and SEM (Fig. 2a and b). The hydrodynamic diameter of typical PPIAO NPs was 333.13 ± 5.34 nm, the polydispersity index (PDI) was 0.19 ± 0.03, and the surface potential was − 4.98 ± 0.73 mV (Additional file 1: Table S1). PPIO NPs have a smooth surface with an average particle size of 313.90 ± 7.28 nm and a surface potential of − 14.60 ± 1.75 mV. Aluminum hydroxide was embedded and adsorbed on the shell of PPIAO NPs mainly through electrostatic attraction, which was the main reason for the change of the surface potential of nanoparticles (Additional file 1: Fig. S1). The encapsulation of aluminum hydroxide in PPIAO NPs was confirmed by FTIR and XPS analysis. The results showed that the two peaks at 564.19 and 556.59 correspond to the Al-O, and the four peaks at 1088.24, 950.76, 1384.37, and 1384.32 correspond to the –OH (Fig. 2c). The characteristic peaks (Fig. 2d) of Al2p and Pt 5p3 binding energy in the spectrum indicated that aluminum (Al) and platinum (Pt) elements were involved in the formation of PPIAO NPs. In addition, the main elements oxygen (O), carbon (C), Al and Pt in PPIAO NPs were also presented in the element mapping (Fig. 2e). The encapsulation efficiency (EE) of aluminum hydroxide in the nanoparticles was 10.4%, and the loading efficiency (LE) was 0.19%. The aluminum content of PPIAO NPs (0.208 µg/mL) was under the safety limit standards for aluminum adjuvants (WHO and European Union 1.25 mg/dose, United States 0.85 mg/dose, China 0.35–3.00 mg/mL) [38–40]. The EE of OXA and ICG in PPIAO NPs was 19.60% and 91.95%. The LE of OXA and ICG in PPIAO NPs was 1.05% and 2.49%. The UV–Vis absorbance spectra and fluorescence spectra of nanoparticles showed a maximum fluorescence emission peak at 780 nm, indicating that ICG was encapsulated into PPIAO NPs without significant changes in its optical properties (Fig. 2f and g).

As a biocompatible near-infrared fluorescent dye, ICG has photothermal, photosensitive, and sound sensitivity properties [41]. Reactive oxygen radicals (ROS) are critical toxic substances in photoacoustic dynamic therapy. SOSG fluorescent probe detected a large amount of singlet oxygen (^1^O_2_) generation after 808 nm laser and ultrasonic irradiation of PPIAO NPs (Fig. 2h). Furthermore, the temperature of the PPIAO NPs aqueous solution gradually increased with increasing of the 808 nm laser intensity under the monitoring of the infrared thermal imager (Fig. 2i). The temperature rose to 62.4 °C after 2 min of 808 nm laser irradiation at 2.5 W/cm^2^ (Fig. 2j). The critical point of lethal temperature for tumor cells is 42.5 to 43 °C, while that for normal cells is 45 °C [42]. Excessive temperature will lead to irreversible denaturation and damage of protein antigens in tumor cells. Therefore, 2 W/cm^2^ was the optimal therapeutic laser intensity. There was no significant difference in temperature change between PPIO NPs and PPIAO NPs (Fig. 2k). The average particle size of PPIAO NPs did not change significantly in DMEM, ddH_2_O, and PBS within 28 days. The fluorescence intensity and UV absorption value of ICG in PPIAO NPs decreased by 20.01% and 17.90%, which are lower than that of free ICG (43.58% and 60.81%) (Additional file 1: Fig. S2). These results suggested that the nanostructures can help ICG maintain optical stability and extend the half-life period.

### PA/ultrasound dual-modality imaging

PFP and ICG enabled PPIAO NPs to have ultrasound and PA imaging capabilities, which were first validated in an in vitro gel model. In both ultrasound and PA imaging, the nanoparticle groups showed signal enhancement after 808 nm laser irradiation (Additional file 1: Fig. S3a). Interestingly, the PA value of the PPIAO group was higher than that of the PPIO group (without Al(OH)_3_) and free ICG group after 808 nm laser irradiation (*p* < 0.05). The photoacoustic signal in Al(OH)_3_ group may be due to the metal ions in aqueous solution. Studies have shown that the optical properties of Fe_3_O_4_ can also be used to enhance photoacoustic molecular imaging [43–45].

**Fig. 3:**
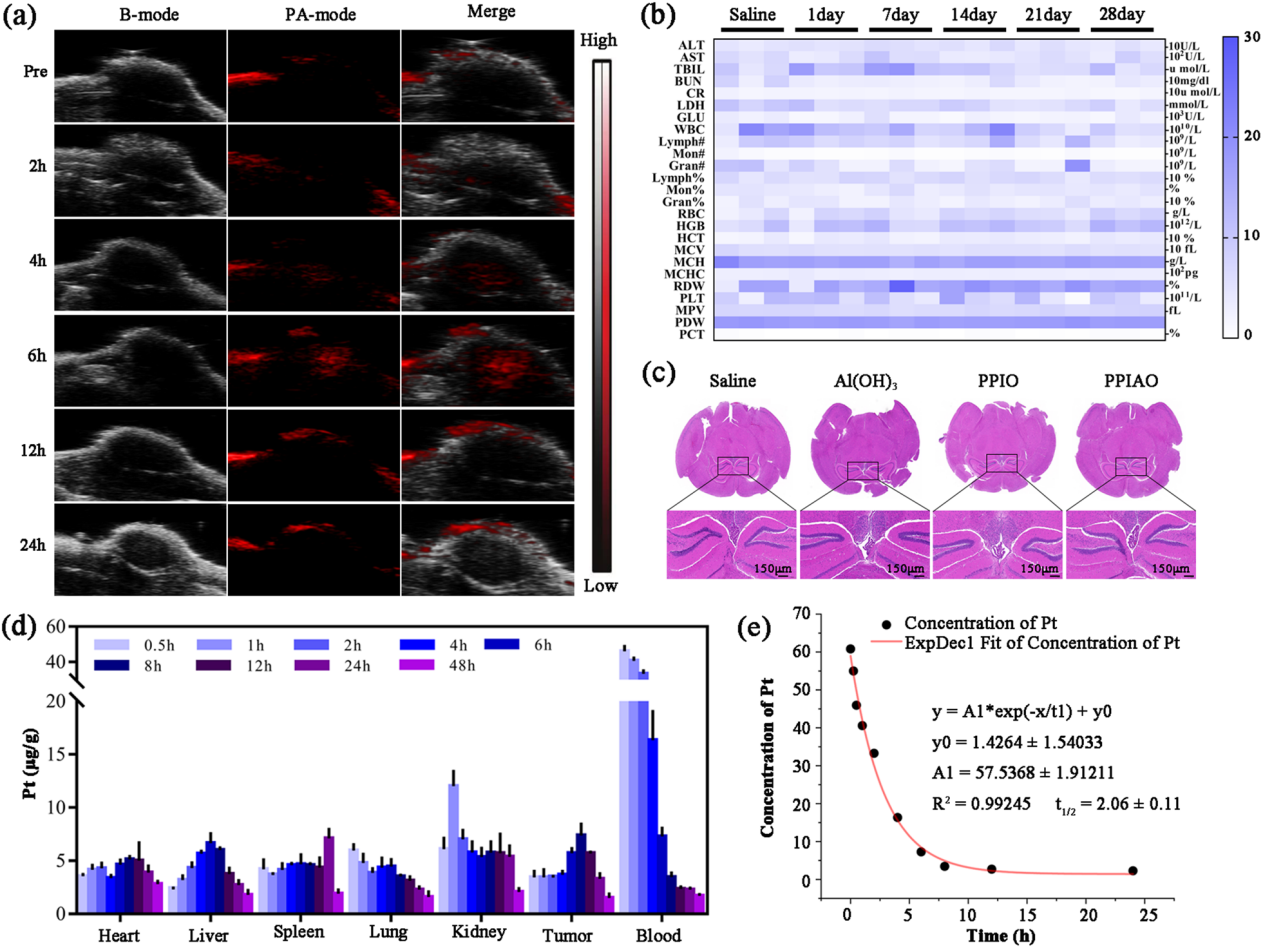
PA imaging, biosafety, pharmacokinetics and biodistribution studies of PPIAO NPs. **a** Tumor ultrasound and PA imaging at different times after injection of PPIAO NPs. **b** Blood cell, blood glucose, hepatic function and renal function tests of PPIAO NPs-administered mice. **c** HE staining of mouse brain tissue. Scale bar 150 μm. **d** Biodistribution of PPIAO NPs. **e** Drug concentration-time profiles of Pt

After an additional ultrasound, the echo intensity (EI) of the PPIAO group decreased by 82.6% in B mode and 68.82% in CEUS mode (Additional file 1: Fig. S3b–e). The photothermal effect accelerated the phase transition of PFP from the liquid to the gas phase in the nanoparticle core. This change increased the acoustic impedance difference between the nano contrast agent and the surrounding environment. The EI and PA values of the nanoparticle group decreased after ultrasonication. This suggested that the phase-transformed nanoparticles were broken under ultrasonication. The results indicated that the nanostructure was crucial for maintaining the optical stability of ICG. Encouraged by the in vitro imaging results, we further explored the potential of PPIAO NPs as ultrasound and PA contrast agents in tumor-bearing mice. Ultrasound and PA images of mouse tumor sites were collected before (Pre), and 2, 4, 6, 12, and 24 h after intravenous administration. As shown in Fig. 3a, the PA signal in the tumor tissue appeared 2 h after administration and peaked at 6 h. This result not only defined the optimal time for in vivo treatment and imaging studies, but also demonstrated the promising performance of PPIAO NPs as PA contrast agents.

### Laser/ultrasound responsive OXA release

The OXA release efficiency of PPIAO was 91.2% and PPIO was 87.5%. Photothermal and ultrasound synergistically destroyed the structure of the nanoparticles, allowing the release of the encapsulated drug. Nanocarriers can not only alter the original distribution and metabolism of drugs, but can also be combined with targeting technology to get drugs to the specified site. Without laser and ultrasound irradiation, OXA release from the nanoparticles was slow (7-day release efficiency < 13.8%), depending on the stability of the nanoparticles.

### Biosafety

Ideal multifunctional nanoparticles for imaging-guided tumor therapy should have the highest possible biosafety and early visualization of tumors, and effective delivery of drugs with reduced side effects. Compared with free OXA, the same concentration of OXA reduced its cytotoxicity due to the encapsulation of nanoparticles (Additional file 1: Fig. S4a). The IC_50_ of cell viability for free OXA was 42.46 µg/mL (Additional file 1: Fig. S4b). The biosafety of PPIAO NPs was further evaluated in healthy C57BL/6 mice. The results of blood cell analysis, blood glucose, hepatic function and renal function tests also showed that the PPIAO NPs at the therapeutic concentration did not cause apparent health damage to the mice within 28 days (Fig. 3b).

Size and surface potential affect the biological safety, distribution and transport process of nanomaterials. Studies have shown that the toxicity of nanomaterials increases with the decrease of particle size (< 100 nm), while oversized particles (> 10 μm) have the risk of microvascular embolism [46–48]. HE staining results showed no obvious histopathological changes in the main organs of the mice (Additional file 1: Fig. S5). And the nano Al(OH)_3_ of PPIAO NPs did not have a significant effect on the mouse brain within 90 days (Fig. 3c). However, the effects of PPIAO NPs on the nervous system and brain function of mice and the evaluation of biosafety in humans need to be further studied.

### Pharmacokinetics and biodistribution studies

Pharmacokinetic and biodistribution studies of intravenously injected PPIAO NPs were performed in ID8 tumor-bearing mice. The in vivo distribution of PPIAO NPs was observed by Pt detected by ICP-MS. The results showed (Fig. 3d) that the peak uptake in tumors of PPIAO NPs reached 7.37 ± 0.88 µg at 6 h after intravenous injection. Pt concentration in blood fit a two-compartment model with time-dependent changes. The half-life of blood circulation was 2.06 ± 0.11 h (Fig. 3e). These results suggested that PPIAO NPs can be safely used as nanotherapeutic agents for in vivo imaging and therapy.

### Synergistic therapeutic efficacy and antigen exposure in Vitro

**Fig. 4:**
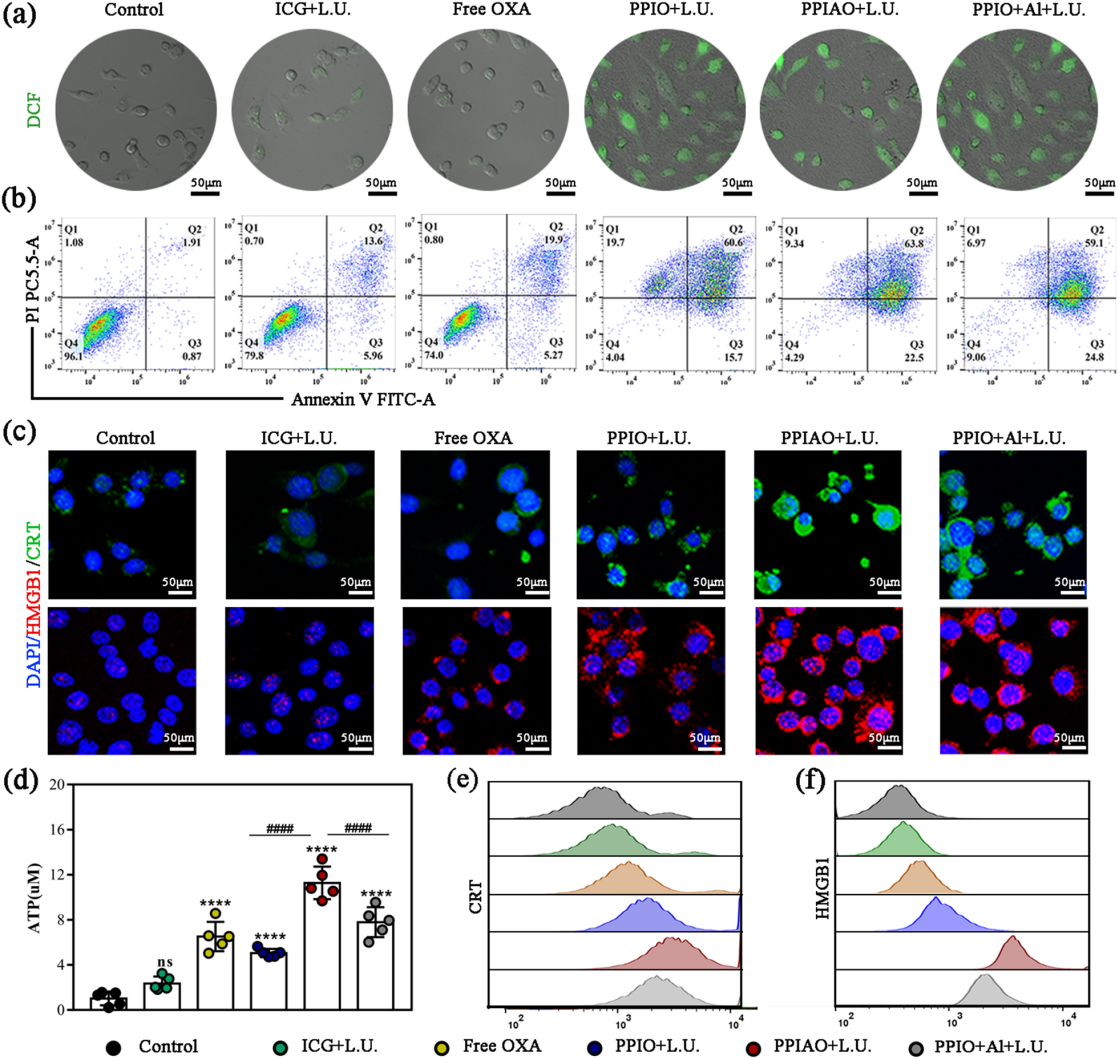
Synergistic therapeutic efficacy and antigen exposure in vitro. **a** ID8 cells were incubated with PPIAO NPs for 2 h and then treated with an 808 nm laser and ultrasound. ROS generation detected by DCFH-DA. CLSM image, scale bar 50 μm. **b** FCM analysis of cell death and apoptosis. **c** CRT membrane inversion and nuclear exocytosis of HMGB1 under CLSM. Scale bar 50 μm. **d** ATP release from ID8 cells (n = 5). **e**,** f** FCM analysis of ID8 cells’ CRT and HMGB1 exposure

Efficient intracellular uptake of PPIAO NPs is a prerequisite for eradicating cancer cells. The 3D image (Additional file 1: Fig. S6a) showed that the PPIAO NPs could successfully enter the cell spheroids and stably aggregate. The phagocytosis rate of tumor cells detected by FCM was 96.3% (Additional file 1: Fig. S6b and c). As expected, tumor cells with phagocytosed nanoparticles showed an increase in intracellular ROS after treatment with 808 nm laser and ultrasound irradiation (Fig. 4a). FCM and CAM/PI stain results showed that the nanoparticles irradiated with 808 nm laser and ultrasound induced complete apoptosis and necrosis (95.96% in PPIO + L.U. group, 95.71% in PPIAO + L.U. group, and 90.94% in PPIO + Al + L.U. group) (Fig. 4b and Additional file 1: Fig. S7a). The results showed that there was no significant difference in the apoptosis rate and necrosis rate among groups PPIO + L.U. group, PPIAO + L.U. group and PPIO + Al + L.U. group. The necrosis rate in the nanoparticle groups did not exceed 10%. And the average apoptotic rate was 87%. In the absence of immune cells and immunoactivity substances, the ability of nanoparticles to promote ID8 cell death was limited (Additional file 1: Fig. S7b). The addition of nano aluminum hydroxide did not contribute significantly to tumor cell killing in vitro. There was no significant difference in cell viability between PPIO + L.U. group (45.24 ± 3.56%) and PPIAO + L.U group (42.10 ± 1.31%) (ns, *p* > 0.05) (Additional file 1: Fig. S7c).

Studies have reported that OXA and ICG-encapsulated nanoparticles can kill tumor cells and trigger ICD simultaneously [21, 22, 31]. It was also validated in the present study, which was manifested by the CRT membrane inversion and HMGB1 nuclear exocytosis and ATP secretion (Fig. 4c and d). In normal cells, CRT was mainly localized in the endoplasmic reticulum and rarely distributed in the cell membrane and nucleus. HMGB1 is mainly located in the nucleus as shown in the control group. CRT rapidly transferred from the endoplasmic reticulum to the cell membrane surface during the early stages of ICD, as demonstrated by solid green fluorescence on dying tumor cells in the PPIAO + L.U. group. In addition to being recognized and bound by DCs as an “eat me” signal, membrane CRT can interact with thrombin and complement C1q to enhance the recognition and phagocytosis of tumor cells by DCs [49]. HMGB1 released from dying tumor cells promotes interaction with Toll-like pattern recognition receptor (TLR-4) on DCs, leading to potent immunostimulatory effects [50]. ATP released by apoptotic tumor cells is a “find me” signal and recruited phagocytes through P2Y(2) receptors on the surface of phagocytes and promoted phagocytosis and clearance of apoptotic cells [51]. Meanwhile, the FCM results also indicated that CRT and HMGB1expressing ID8 cells were detected in the PPIAO + L.U group (Fig. 4e and f). The above results indicated that the combination therapy with PPIAO NPs could simultaneously annihilate tumor cells and expose tumor antigens. Other specific antigens or tumor-associated antigens released by tumor cells after combination therapy have not been precisely defined. But, they are also essential for the activation of APCs and T lymphocytes.

### Antigen capture and DC stimulation in vitro

Sufficient exposure to tumor antigens is necessary for inducing a potent antitumor immune response. Aluminum salt adjuvants delay antigen degradation and prolong the retention time of antigen by changing the physical properties of antigen, which stimulates APCs to enhance their processing and presentation of antigen. Studies have shown that nanoparticles with smaller particle sizes as antigen carriers have stronger adjuvant activity. Compared with conventional aluminum hydroxide particles of approximately 9.3 μm, the adsorption of the same protein antigen on nano aluminum hydroxide can induce stronger antigen-specific responses [52]. The protein capture amount of PPIAO NPs loaded with aluminum hydroxide nanoparticles was 551.67 µg/mL, which was significantly different from the PPIO NPs group without nano aluminum (*p* < 0.001) (Additional file 1: Fig. S8a and b). After capturing protein antigens, the average particle size of nanoparticles increased, and the surface potential decreased (Additional file 1: Fig. S8c and d). Membrane-like substances appeared on the surface of the aluminum hydroxide nanoparticles after incubation with BSA (Additional file 1: Fig. S9a). To determine whether the capture protein contained TAAs, the captured proteins were compared with tumor cell lysates by gel electrophoresis. The results showed differences in protein profiles (Additional file 1: Fig. S9b).

**Fig. 5:**
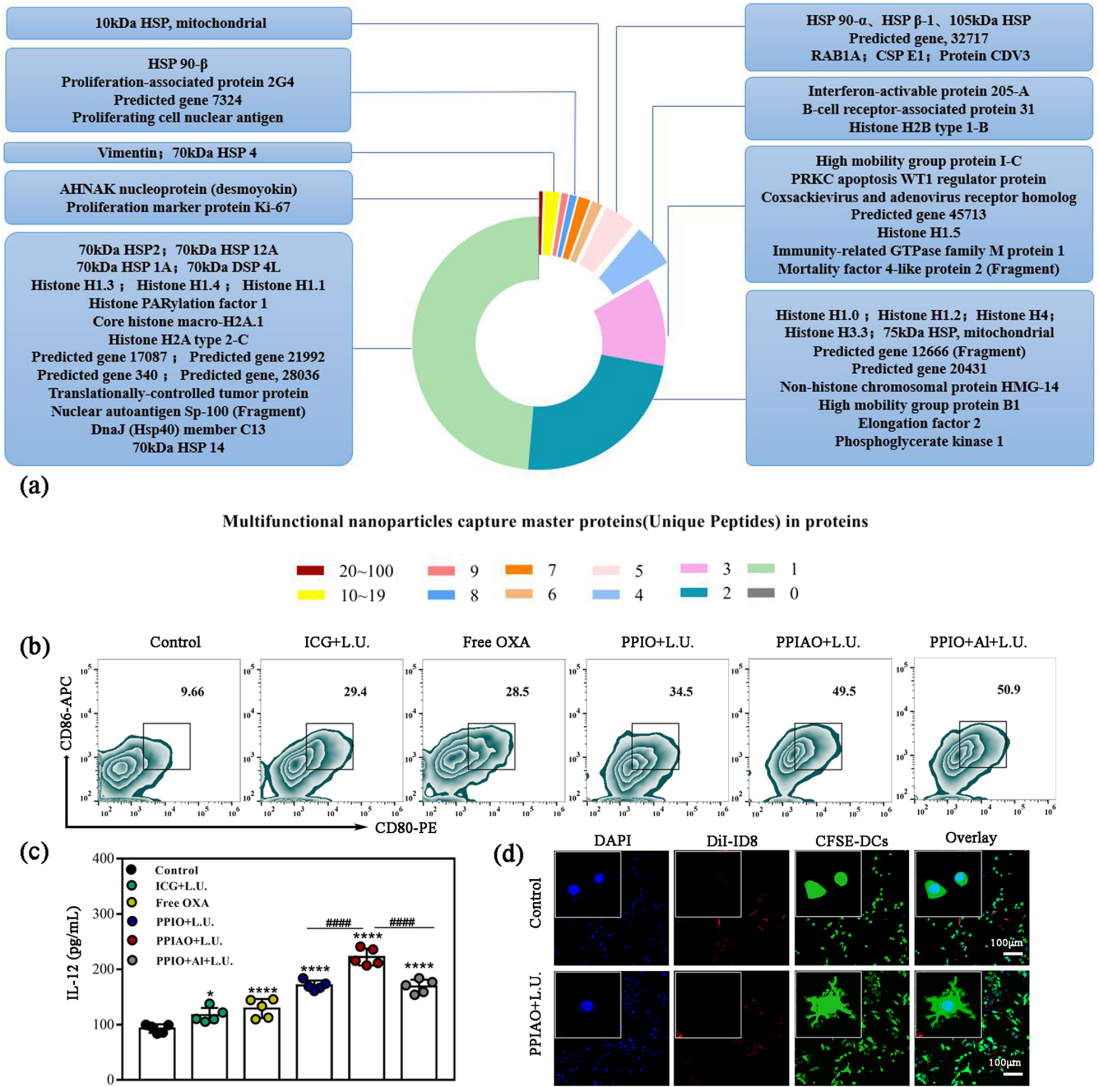
Antigen capture and DC stimulation. **a** Tumor antigens and predicted genes in the captured protein detected by LC/MS/MS (stratified by the number of specific peptides contained in the protein). **b** DC maturation was detected by FCM. **c** IL-12 secretion levels in different groups were detected by ELISA (n = 5). **d** Typical morphology change of DC cells in the control group and PPIAO + L.U. group. Scale bar 100 μm

Tumor antigens in the capture protein were further confirmed by LC/MS/MS analysis. Proteins with a Sum PEP Score ≥ 1.5 were screened out, and contaminating proteins were deleted. There were 2632 main proteins containing specific peptides in the capture protein, including 2.85% of known TAAs, such as PGK1, AHNK, RPSA, HNRPL, RPS2, RPL10A, nine predicted genes, and many functional proteins related to tumor immune metabolism (Fig. 5a). The first stage of the antitumor immune response involves the recognition, processing, and presentation of antigens by APCs. DCs are critical initiators of adaptive immune responses and activate T cells by capturing and cross-presenting antigens released by tumor cells [53]. DC maturation was identified by costimulatory molecule expression, cytokine secretion, and cell morphology. The results of FCM showed that DC maturity (CD11c^+^ CD86^+^ CD80^+^) increased in the ICG + L.U. group (3.04 fold) and free OXA group (2.95 fold) compared with the control group, while the PPIAO + L.U. group was more significant (5.12 fold) (Fig. 5b). IL-12 is a cytokine secreted by mature DCs, that induces naive T cells (Th0) to differentiate into Th1 cells and promotes a Th1-type immune response. The results showed a higher level of secretion in the PPIAO + L.U. group (*p* < 0.0001) (Fig. 5c). The DCs in the PPIAO + L.U. group showed typical dendritic protrusions under CLSM, which are immature DCs (iDCs) that gradually became mature DCs (mDCs) after ingesting antigens (Fig. 5d). We observed the internalization of nanoparticle-captured antigens by APCs in an independent experiment. OVA-FITC-adsorbed DiI-labeled PPIAO NPs (DiI-NPs) were coincubated with DCs and macrophages. In addition to the colocalization of red and green fluorescence in the membrane and cytoplasm of both cells observed in the CLSM images, we also found a separate green fluorescence, which may be the APC uptake of OVA-FITC adsorbed by aluminum hydroxide (Additional file 1: Fig. S10). In conclusion, PPIAO NPs irradiated with 808 nm laser and ultrasound achieved sufficient exposure to tumor antigens. The tumor antigens captured by nano aluminum hydroxide effectively induced DC maturation and promoted antigen presentation, which is beneficial for activating an antitumor immune response.

### In vivo antitumor therapeutic effect

**Fig. 6:**
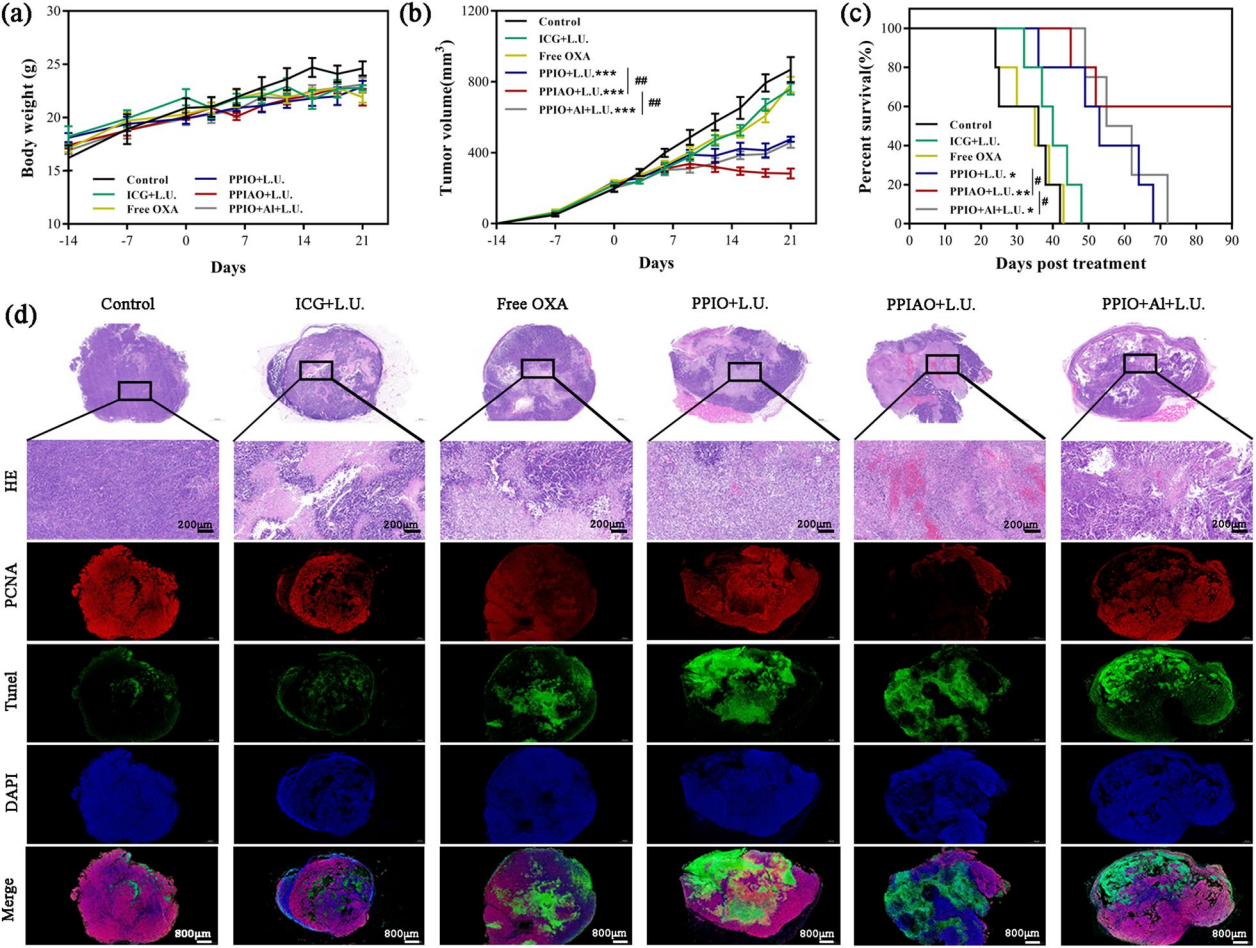
In vivo antitumor therapeutic effect. **a**,** b** Changes in body weight and tumor volume of mice (n = 5). **c** Survival curves of mice in each group (n = 5). **d** HE/PCNA/TUNEL immunohistofluorescence staining of tumor tissue sections. HE, scale bar 200 μm. PCNA/TUNEL, scale bar 800 μm

The efficacy of PPIAO NPs mediated combination therapy was evaluated by subcutaneous transplantation of ID8 ovarian cancer in female C57BL/6 mice (Additional file 1: Fig. S11a). By measuring the tumor volume after different treatments and the survival time of mice, we proved that PPIAO NPs mediated combination therapy plays an important role in inhibiting tumor growth in vivo. During the observation period, there was no significant difference in the body weight of the mice in each group (*p* > 0.05) (Fig. 6a). Compared with the ICG + L.U. group and free OXA group, the tumor growth of the mice in the nanoparticle combined L.U. groups were inhibited (*p* < 0.001) (Fig. 6b). Survival statistics also showed that they achieved more prolonged survival than the control and monotherapy groups after reducing their tumor burden by combination therapy (*p* < 0.001) (Fig. 6c). Based on the results of biosafety studies of nanoparticles in vitro and in vivo, PPIAO NPs alone had no obvious inhibitory effect on the growth of ID8 cells and no significant damage to the health of mice. PPIAO NPs alone group was not included in the control group in the study of antitumor effects for the ethical requirements of experimental animals.

The underlying mechanism of drug-loaded nanoparticles mediated by near infrared and ultrasound has not been fully clarified yet. We propose that the improvement of anticancer efficiency after combined therapy may be caused by many factors. ICG mediated PTT/PDT was the leading cause of tumor cell death. Thermal infrared imaging showed that the temperature at the tumor site was approximately 55 °C during the treatment (Additional file 1: Fig. S11b and c). Heat destroys new tumor blood vessels and causes thermal damage to tumor cells [54]. PDT increases intracellular ROS and phototoxic chemicals, which can induce direct cytotoxicity and local microvascular damage [24]. Secondly, the mechanical damage of the cell membrane caused by the cavitation effect of ultrasound and the rupture of phase change nanoparticles promoted the release of O_2_ and OXA [32]. Our previous studies have shown that improving hypoxia in the tumor microenvironment increases the sensitivity of chemotherapy to tumors [55]. Our results also showed that the nanoparticle combined L.U. groups had a greater extent of tissue destruction, tumor cell apoptosis and proliferation inhibition (Fig. 6d). In addition, compared with the PPIO + L.U. group, the mice in the PPIAO + L.U. group survived longer, which may be due to the tumor antigen trapping function and adjuvant effect of Al(OH)_3_ [27, 28].

### Antigen exposure and antitumor immune response induction in vivo

**Fig. 7:**
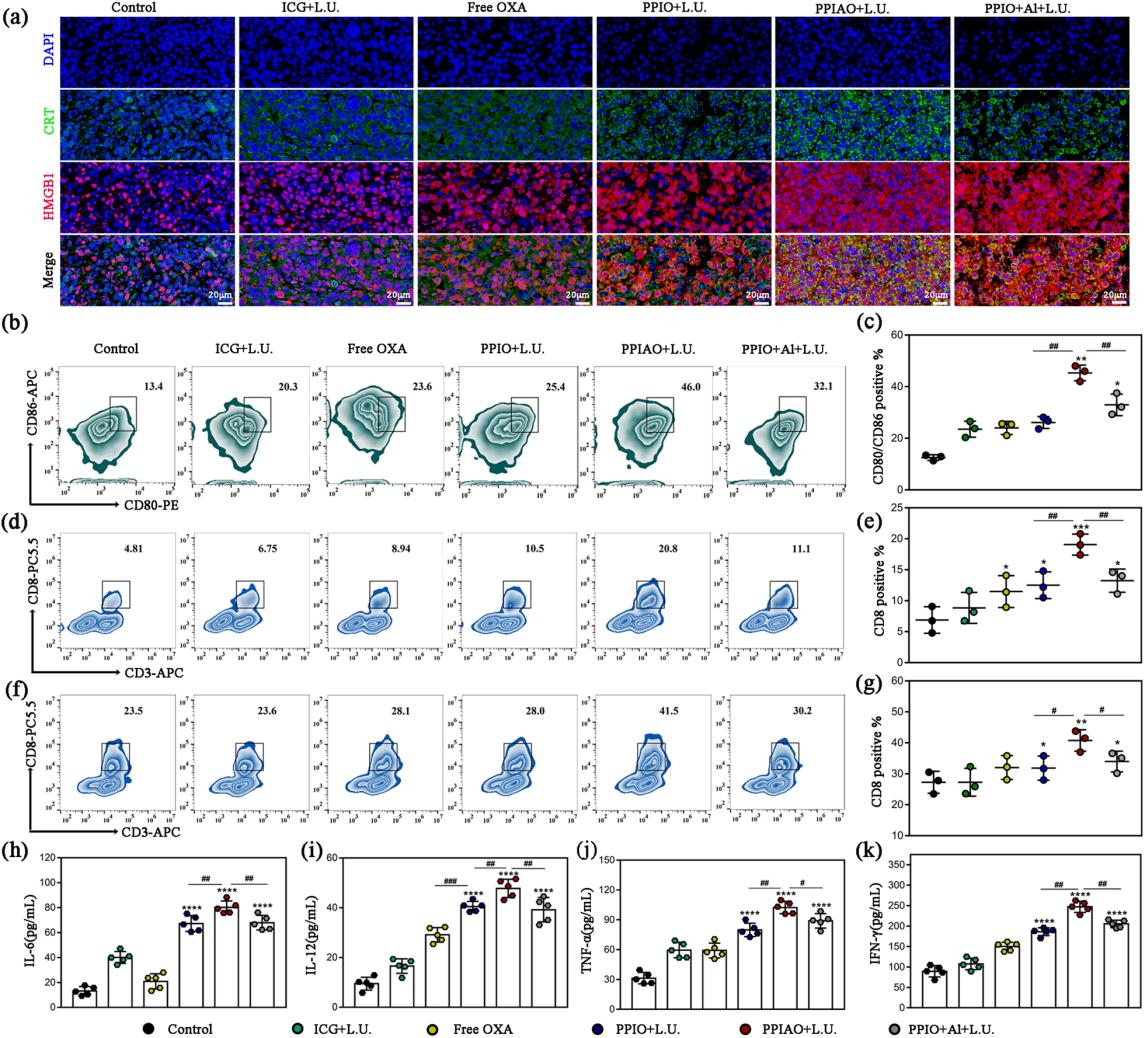
Antigen exposure and antitumor immune response induction in vivo. **a** Immunofluorescence staining of intratumoral CRT/HMGB1. Scale bar 20 μm. **b**,** c** Intratumoral mature DC abundance detected by FCM. **d**,** e** Intratumoral activated CD8^+^ T lymphocytes abundance was detected by FCM. **f**,** g** The activation of splenic CD8^+^ T lymphocytes was detected by FCM. **h–k** The secretion levels of IL-6, IL-12, TNF-α, and IFN-γ in the serum of mice after 7 days of treatment were detected by ELISA (n = 5)

The expression of ICD related molecules in intratumoral cells was observed by immunofluorescence (Fig. 7a). Almost all intratumoral cells in the PPIAO + L.U. group exhibited membrane inversion of CRT and extracellular secretion of HMGB1. This was completely different from the CRT cytoplasmic hypofluorescence signal and nuclear colocalization of HMGB1 presented in the control group. Theoretically, PPIAO + L.U. treatment can induce the exposure of other tumor antigens that cannot be confirmed and even the expression of neoantigens as in vitro studies. Neoantigen vaccines promote stronger T cell-specific immune responses and lead to antigen spread [53]. There was a difference in the mean fluorescence intensity between the combination treatment groups. However, whether these differences are attributable to nano aluminum hydroxide requires further verification. Tumor antigens and risk-associated molecular patterns released by tumor cells promote DC maturation, antigen uptake processing, and presentation between MHC-I and MHC-II molecules (via antigen cross-presentation) [56, 57]. Intratumoral mDCs (CD11c^+^ /CD80^+^ /CD86^+^) were detected by FCM. The highest abundance of mDC was found in PPIAO + L.U. group probably due to the critical role of nano aluminum hydroxide in tumor tissue (Fig. 7b and c). Aluminum hydroxide adsorbs and stores antigens and converts soluble antigens into particulate antigens. Studies have suggested that the slow release of antigens and increased cellular recruitment may not be related to the adjuvant properties of aluminum. The retention of antigens at the injection site by aluminum hydroxide promoted the uptake of particulate antigens by mature migrating DCs 24 h later [25, 26]. In secondary lymphoid organs, such as the spleen or lymph nodes, these DCs interact and activate naive T lymphocytes via MHC-T cell receptor recognition and coreceptors. Cytotoxic T lymphocytes (CTLs) are the main force in mediating specific anti-tumor cellular immunity. The highest abundance of CD8^+^ T cells appeared in the spleen and tumor tissues of mice in the PPIAO + L.U. group (Fig. 7d–g and Additional file 1: Fig. S12). Furthermore, successful antitumor immunity requires tacit cooperation between immune and nonimmune components. In particular, IFN-γ has a strong immunoregulation function and promotes cellular immunity. Mice in PPIAO + L.U. group developed higher levels of cytokines (IL-6, IL-12, TNF-α, and IFN-γ) in serum (Fig. 7 h–k).

### Abscopal effect and in situ tumor vaccines effects

**Fig. 8:**
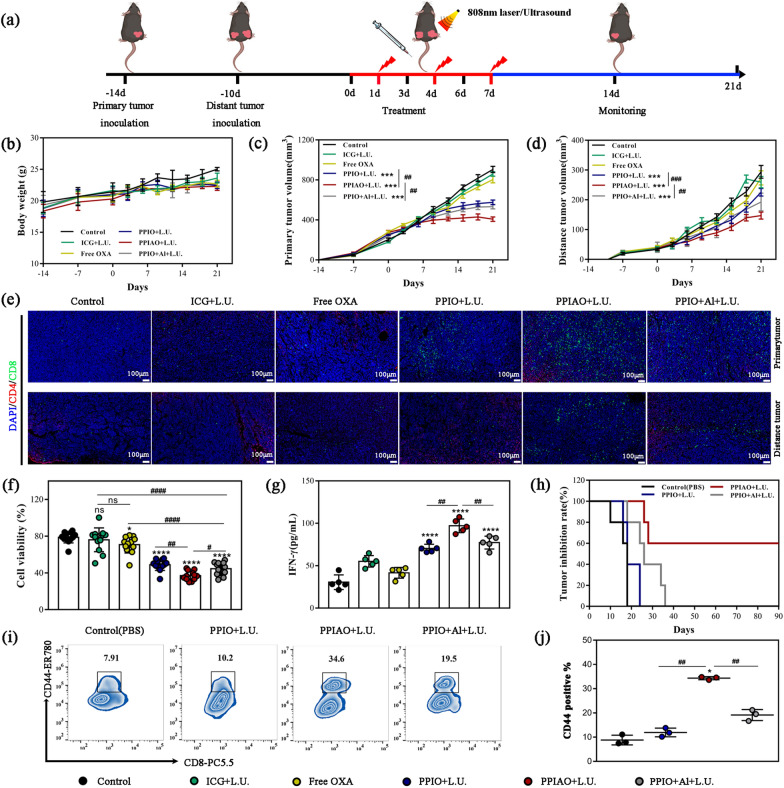
Abscopal effect and in situ tumor vaccine effects. **a** PPIAO NPs combined with 808 nm Laser and ultrasound treatment inhibited distant tumor growth in vivo. **b** Body weight of mice in each group (n = 6). **c**,** d** Primary tumor volume and distance tumor volume of mice (n = 6). **e** Immunofluorescence of CD4^+^ T/CD8^+^ T lymphocytes in primary tumors and metastases of mice. Scale bar 100 μm. **f** ID8 tumor cell viability cocultured with spleen lymphocytes (n = 11). **g** Detection of IFN-γ secretion in spleen lymphocytes of mice by ELISA (n = 5). **h** Female C57BL/6 mice were inoculated with differently treated ID8 tumor spheroids. Tumor rate in each group after ID8 cell rechallenge (n = 5). **i**,** j** Detection of CD3^+^/CD8^+^/CD44^+^ T memory lymphocyte abundance in the spleen of vaccine mice by FCM

To further elucidate the mechanism of the favorable tumor suppressive effect, we established a bilateral tumor model to evaluate immune cell responses to metastatic tumors (Fig. 8a). One mouse in the control group died due to excessive tumor burden, while the changes in body weight in the other groups were not abnormal during the observation period (Fig. 8b). Compared with the monotherapy groups, primary tumor growth was significantly inhibited in the combination therapy groups (36% in PPIO + L.U. group, 65% in PPIAO + L.U. group, and 41% in PPIO + Al + L.U. group) (*p* < 0.001). Compared with the PPIO + L.U. group and the PPIO + Al + L.U. group, the mice in PPIAO + L.U. group had the smallest tumor volume (*p* < 0.01) (Fig. 8c). This difference in growth inhibition was more pronounced in metastatic tumors (23% in PPIO + L.U. group, 50% in PPIAO + L.U. group, and 34% in PPIO + Al + L.U. group). The mean volume of metastatic tumors in PPIAO + L.U. group was significantly smaller than that in PPIO + L.U. group (*p* < 0.001) and PPIO + Al + L.U. group (*p* < 0.01) (Fig. 8d). Five mice failed to present with metastases in PPIAO + L.U. group on day 21 (Additional file 1: Fig. S13). T cells are emerging as pivotal regulators of antitumor immunity and determinants of the immunotherapy response. Activated CD8^+^ T cells can eliminate ID8 tumor cells via perforin and gamma interferon and return to the tumor microenvironment through cognate interactions to control tumor growth. CD4^+^ T cells are integral to CD8^+^ T cell activation and tumor immunity [58]. Seven days after primary tumor treatment, more CD8^+^ T cells and CD4^+^ T cells were distributed in both the primary tumor and the metastatic tumor of the PPIAO + L.U. group (Fig. 8e). Meanwhile, the abundance of activated CD8^+^ T lymphocytes in spleen lymphocytes was highest in PPIAO + L.U. group (Additional file 1: Fig. S14). According to these results, PPIAO NPs mediated combination therapy could induce T cells to differentiate into CD8^+^ T cells and increase CD8^+^ T cell infiltration into tumors significantly. In addition, to evaluate the systemic efficacy based on PPIAO + L.U. therapy, spleen lymphocytes from mice were cocultured with ID8 cells. The tumor cell activity in the PPIAO + L.U. group was the lowest (37.02 ± 4.86%), which was significantly different from the other groups (*p* < 0.05) (Fig. 8f). The highest concentration of IFN-γ was detected in the cell supernatant of the PPIAO + L.U. group by ELISA (Fig. 8g). IFN-γ is mainly produced by activated NK cells and T cells. This suggested that PPIAO NPs combined with laser-ultrasound irradiation activated systemic T cells, which is beneficial for clearing other small tumor metastases in vivo.

To determine whether the primary tumor after treatment has the potential to become an orthotopic tumor vaccine, we inoculated ID8 tumor cell spheroids after combination treatment into the left hind leg of healthy female C57BL/6 mice. The vaccine was given twice, seven days apart. One week after the last vaccination, mice in each group were subcutaneously inoculated with the same batch of cultured ID8 tumor cells on the right back. The tumor formation of mice in each group was then observed. All mice in the control group (PBS) and PPIO + L.U. group had visible palpable tumors 21 days after tumor cell challenge (100% tumor formation rate). There were four tumor-bearing mice (80%) in the PPIO + Al + L.U. group and two tumor-bearing mice (40%) in the PPIAO + L.U. group with smaller tumor volumes (Fig. 8h and Additional file 1: Fig. S15). Within 40 days, the tumor rate of the PPIAO + L.U. group was 40%, while that of the other groups was 100%. No tumor formation was seen in the remaining three mice in the PPIAO + L.U. group during the 90-day observation period.

Vaccine responses benefit from enhanced antigen presentation, migration of dendritic cells within tissues, and increased antigen transport to lymph nodes [16]. Nano aluminum hydroxide in PPIAO NPs promotes the migration of immune cells to lymph nodes by acting as an adjuvant, which exposes more antigens to the immune system [59]. This may be why more mice in the PPIAO + L.U. group resisted tumor cell attack. Memory T cells (Tm) are prominent members of the defense against attack [60]. The expression of CD44 is an indicator of effector-memory T cells. A higher abundance of Tm (CD3^+^/CD8^+^/CD44^+^) activation was detected in the spleen of mice in the PPIAO + L.U. group (Fig. 8i and j). Encouraged by the anticancer outcome after cell vaccination, we attempted to rechallenge ID8 cells in tumor-bearing mice (n = 5) treated with PPIAO + L.U. to observe the vaccine effect on tumor tissue. Until 4 weeks post rechallenge, we observed pink mass formation at the ID8 injection site (left posterior back) in two mice, which represented a tumor appearance. There was no neonatal mass at the injection site of the remaining three mice during the 90-day survival observation period. Compared with spheroids, tumor tissue exhibited better vaccine effects, such as delaying tumorigenesis. The reasons for this result are complex. However, it is worth affirming that PPIAO NPs mediated combination therapy contributed more in inducing tumor antigen exposure, promoting mature DC antigens present, activating T lymphocytes, and increasing intratumoral infiltration. These contributions were crucial for the induction of antitumor immunity and the formation of immune memory in mice (Additional file 2).

In addition to verifying the versatility of combination therapy, the therapeutic effect of PPIAO NPs combined with immune checkpoint suppression (ICI) should be explored further. The molecular mechanism of the interaction between captured antigens and APCs is not clear yet. The uptake of captured antigens by APCs is only the beginning of exploring the fate of tumor antigens in cells. Both the type and molecular weight of antigens affect the absorption pathway of APCs. The transport of captured antigens in the lymphatic system and the presentation of captured antigens to T lymphocytes should be observed in our next work.

## Conclusion

In this study, PPIAO NPs combined with 808 nm laser/ultrasound not only effectively destroyed the primary tumor, but also inhibited metastasis tumor growth by inducing antitumor immunity, which suggested that a nanoplatform design based on tumor antigen capture may be more suitable for personalized immunotherapy. PFP and ICG enabled PPIAO NPs to have ultrasound and PA imaging capabilities. PTT/PDT and OXA promoted tumor cell apoptosis and induced tumor antigen exposure. O_2_ further improved the efficacy of combination therapy by improving the hypoxic microenvironment of the tumor. Al(OH)_3_ timely captured released tumor antigens and promoted tumor immunity. FDA-approved materials are used to synthesize PPIAO NPs, which are more beneficial for future clinical translation. We look forward to these combined strategies based on PPIAO NPs being attempted in the diagnosis and treatment of other highly aggressive tumors. The developed multifunctional tumor antigen trapping nanoparticles may be a promising nanoplatform integrating multimodal imaging monitoring, tumor therapy, and tumor vaccine immunotherapy.

## Supplementary Information

**Additional file 1.** Additional Tables S1, S2 and Figures S1–S15.

**Additional file 2.** In vitro antigen capture validation. SDS-PAGE protein analysis of tumor cell lysate and nano-Al(OH)_3_ capture protein. Samples stained with Coomassie Blue.

## Data Availability

The datasets generated or analyzed during this study are included in this published article.

## Abbreviations

PTT: Photothermal therapy
PDT: Photodynamic therapy
APCs: Antigen-presenting cells
CTLs: Cytotoxic T lymphocytes
ITM: Immunosuppressive tumor microenvironment
ICD: Immunogenic cell death
ATP: Adenosine triphosphate
CRT: Characteristic calreticulin
HMGB1: High mobility group 1
HSP: Heat shock proteins
ABAs: Aluminum-based adjuvants
DCs: Dendritic cells
SEM: scanning electron microscopy
PDI: Polydispersity index
FTIR: Fourier transform infrared spectrometer
XPS: X-ray photoelectron spectroscopy
ICG: Indocyanine green
ROS: Reactive oxygen radicals
OXA: Oxaliplatin
FDA: Food and Drug Administration
EDS: Energy dispersive spectroscopy
SOSG: Singlet oxygen sensor green
HPLC: High-performance liquid chromatography
PAI: Photoacoustic Imaging
EI: Echo intensity
PFP: Perfluoropentane
ICP-MS: Inductively coupled plasma mass spectrometry
CLSM: Confocal scanning microscopy
FCM: Flow cytometry
TLR-4: Toll-like pattern recognition receptor
PCNA: Proliferating cell nuclear antigen
TUNEL: Terminal deoxynucleotidyl transferase-mediated dUTP nick end labeling
CTLs: Cytotoxic T lymphocytes
TME: Tumor microenvironment
ICI: Immune checkpoint inhibition
PVA: Polyvinyl alcohol
DLS: Dynamic laser light scattering
