# Supplementary material for: Photoacoustic mediated multifunctional tumor antigen trapping nanoparticles inhibit the recurrence and metastasis of ovarian cancer by enhancing tumor immunogenicity
Source: J Nanobiotechnology. 2022 Nov 3;20:468. doi: 10.1186/s12951-022-01682-5 (PMC9632083; doi:10.1186/s12951-022-01682-5)
Supplement: Supplementary file 1 — Additional file 1. Additional Tables S1, S2 and Figures S1–S15. [file 12951_2022_1682_MOESM1_ESM.docx]

Additional Information

**Photoacoustic mediated multifunctional tumor antigen trapping nanoparticles inhibit the recurrence and metastasis of ovarian cancer by enhancing tumor immunogenicity**

Xiaowen Zhong^1^, Chenyang Li^1^, Guangzong Zhao^1^, Mengmeng Li^1^, Shuning Chen^1^, Yang Cao^2^, Qi Wang^3^, Jiangchuan Sun^1^, Shenyin Zhu^4*^, and Shufang Chang^1*^

Table S1. Physicochemical properties of the prepared nanoparticles

| NPs | Size  [nm] | Zeta potential  [mV] | PDI | EE% of  (ICG) | LE% of (ICG) | EE% of (OXA) | LE% of (OXA) | EE% of (Al) | LE% of (Al) |
| --- | --- | --- | --- | --- | --- | --- | --- | --- | --- |
| Al(OH)_3_ | 87.77±4.18 | 41.38±1.24 | 0.17±0.01 | _ | _ | _ | _ | _ | _ |
| PPIO | 313.90±7.28 | -14.60±1.75 | 0.15±0.06 | 92.52 | 2.55 | 16.03 | 0.88 |  |  |
| PPIAO | 333.13±5.34 | -4.98±0.73 | 0.19±0.03 | 91.95 | 2.49 | 19.60 | 1.05 | 10.40 | 0.19 |

The equations for encapsulation efficiency (EE) , loading efficiency (LE) are as follows:

$\mathrm{EE}\left( \% \right)=\frac{total mass of added drug - mass of drug in supernatant}{total mass of added drug}\times100\%$ Equation 1

$LE (\%)=\frac{total mass of added drug - mass of drug in supernatant}{total mass of NPs}\times100\%$ Equation 2

$OXA release (\%) =\frac{OXA amount in supernatant}{OXA amount in total package} \times100\%$ Equation 3

**
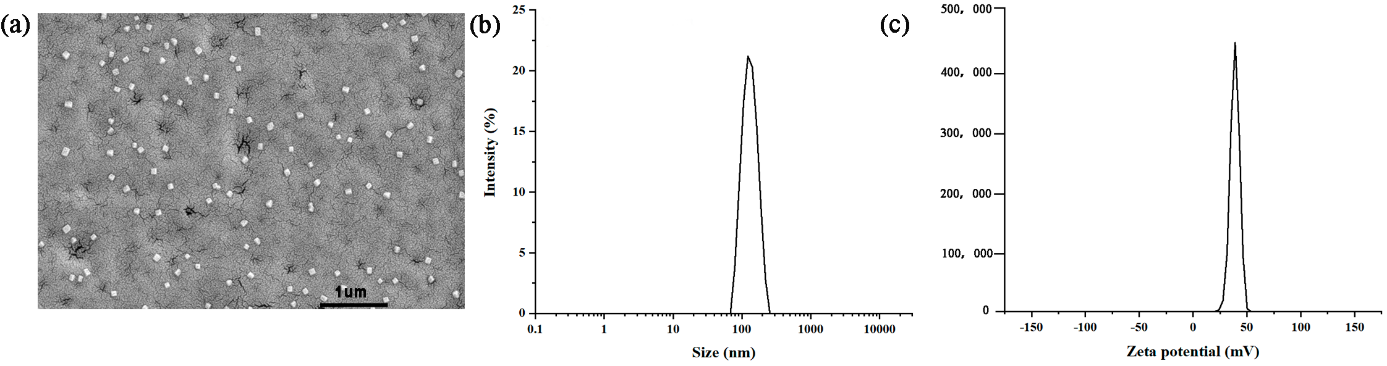
**

Figure S1. (**a**) Scanning electron microscopy of nanoaluminum hydroxide. (**b,c**) The particle size and zeta potential of nanoaluminum hydroxide.

**
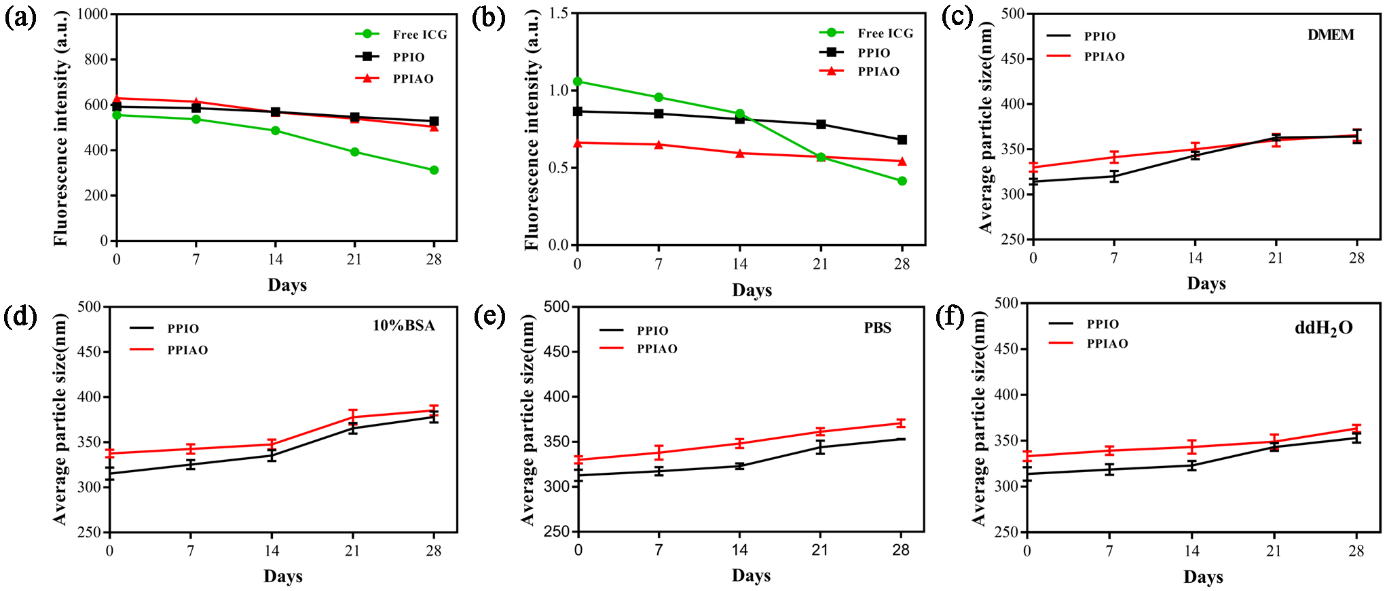
**

Figure S2. (**a**) Fluorescence stability of PPIAO NPs. (**b**) Ultraviolet absorption stability of PPIAO NPs. (**c-f**) Particle size stability of PPIAO NPs in DMEM, 10% BSA, PBS and ddH_2_O.


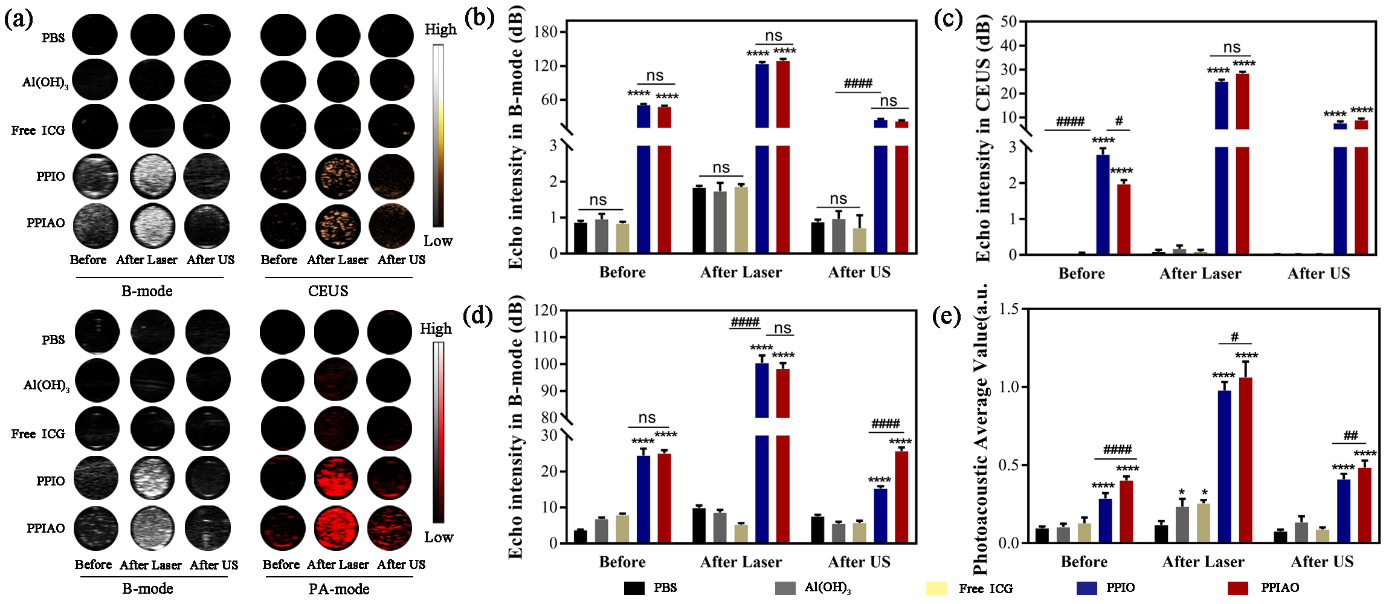


Figure S3. (**a**) Comparison of ultrasound imaging and PA imaging of PPIAO NPs, PPIO NPs, Al(OH)_3_ and free ICG. (**b,c**) EI in B-mode and CEUS during ultrasound imaging (n = 3). (**d,e**) EI in B-mode and average PA value of PA-mode during photoacoustic imaging (n = 3). * Compared with the control group, * *p* < 0.05, ** *p* < 0.01, *** *p* < 0.001, **** *p* < 0.0001; ^#^ was the comparison between groups, ^#^ *p* < 0.05, ^##^ *p* < 0.01, ^###^ *p* < 0.001, ^####^ *p* < 0.0001; ns, *p* > 0.05.

**
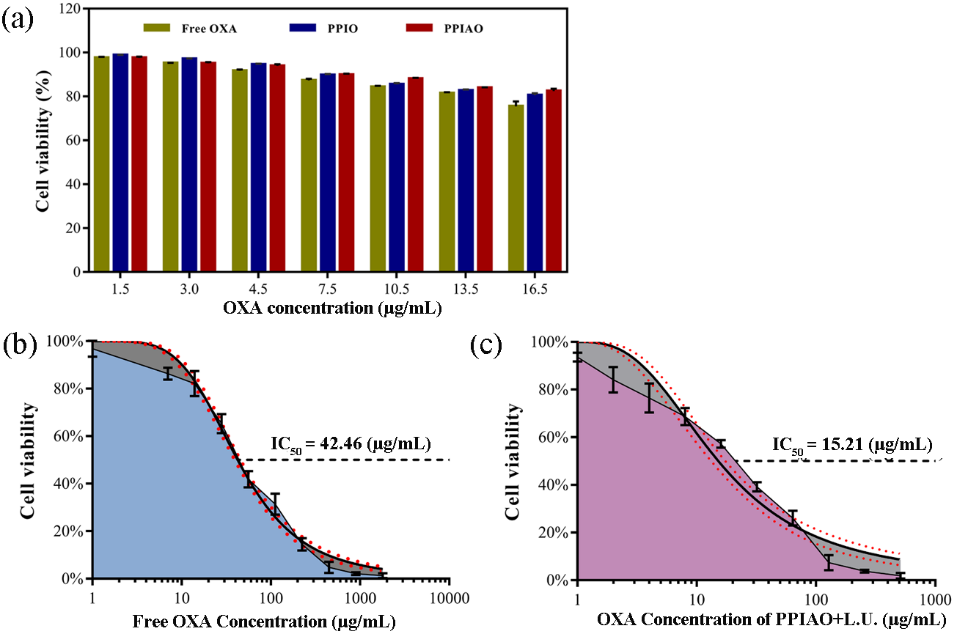
**

Figure S4. (**a**) CCK-8 assays the cytotoxicity of PPIAO NPs, PPIO NPs and free OXA (n = 5). (**b**) The IC_50_ of cell viability for free OXA. (**c**) The IC_50_ of cell viability in the PPIAO + L.U. group.


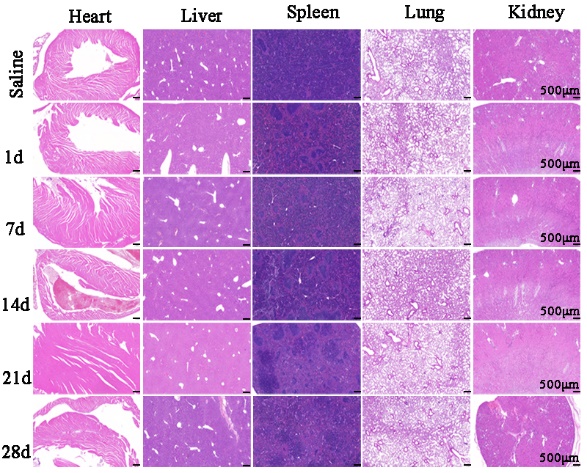


Figure S5. The biosafety of PPIAO NPs was evaluated in healthy C57BL/6 mice. HE staining of the main organs. Scale bar 500 μm.

**
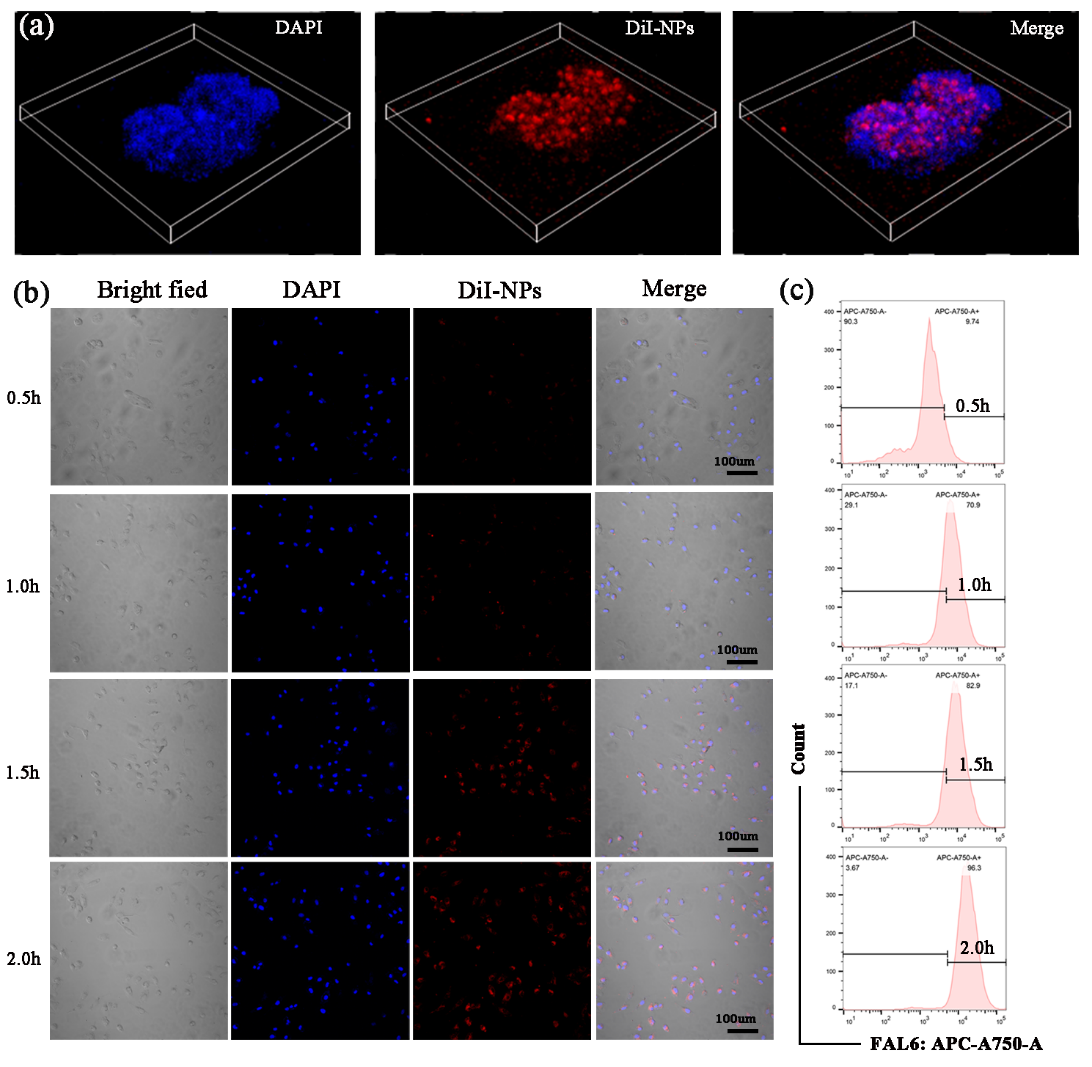
**

Figure S6. (**a**) Infiltration and aggregation of PPIAO NPs in tumor spheroids under CLSM.

(**b**) Phagocytosis of PPIAO NPs by ID8 cells co-incubated for 0.5, 1.0, 1.5, and 2 h. (**c**) The phagocytosis rate of tumor cells detected by flow cytometry.

**
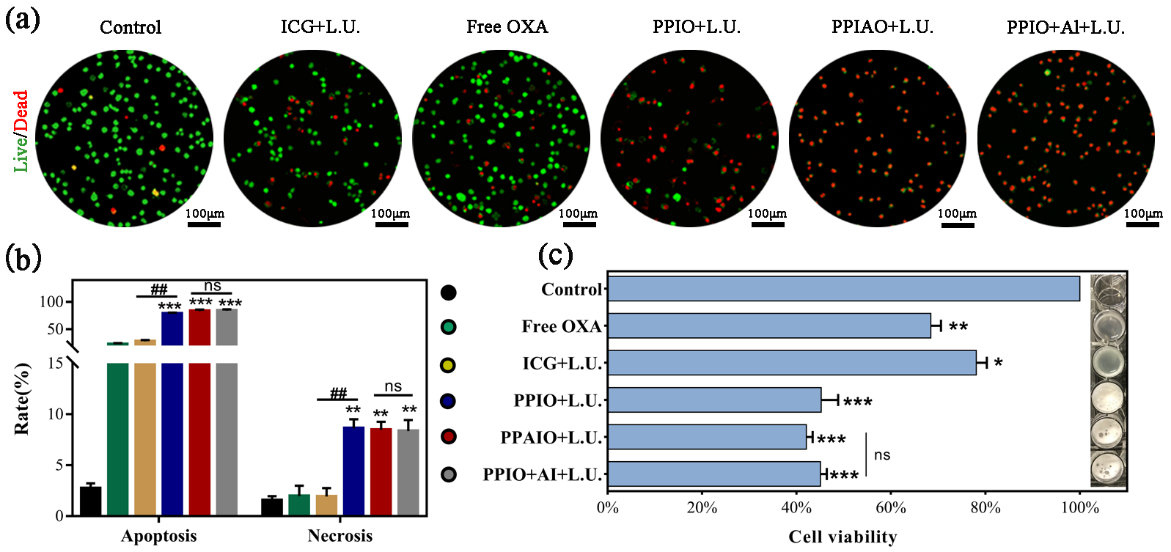
**

Figure S7. ID8 cells were co-incubated with PPIAO-NPs (OXA 15.21 μg/mL) for 2 h, then treated with 808 nm laser (2 W/cm^2^, 5 min) and ultrasound (1 W/cm^2^, 5 min). (**a**) Confocal images of CAM/PI staining of ID8 cells. Scale bar 100 μm. (**b**) The rate of apoptosis and necrosis of tumor cells (n = 5). (**c**) Cell viability was detected by CCK-8 (n = 5). * Compared with the control group, ^*^ *p* < 0.05, ^**^ *p* < 0.01, ^***^ *p* < 0.001, ^****^ *p* < 0.0001; # was the comparison between groups, ^#^ *p* < 0.05, ^##^ *p* < 0.01, ^###^ *p* < 0.001, ^####^ *p* < 0.0001; ns, *p* > 0.05.

**
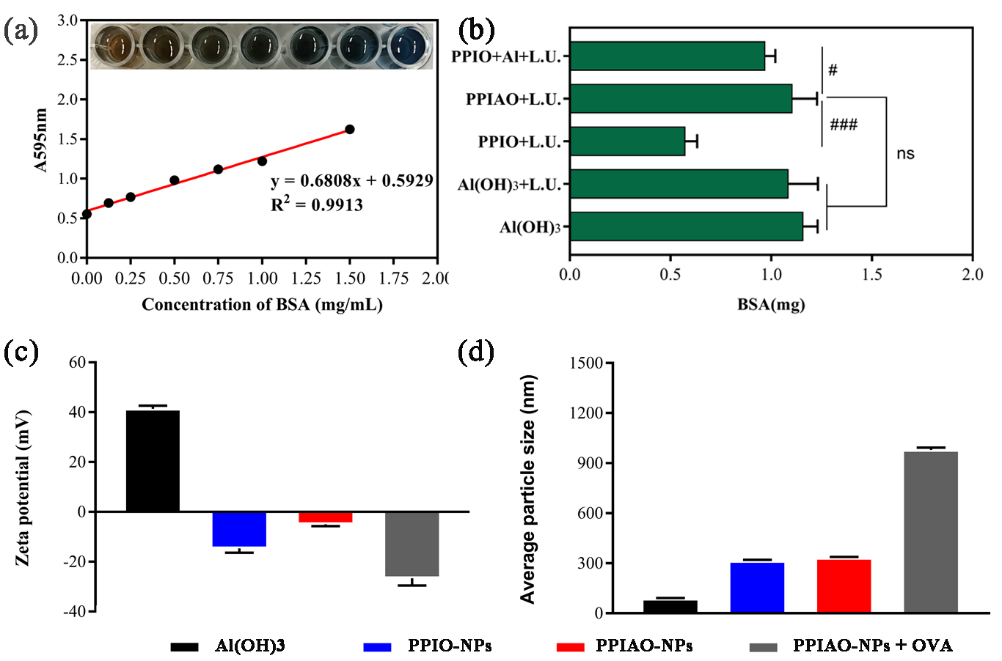
**

Figure S8. (**a**) The concentration scale of BSA detected by Bradford method. (**b**) The adsorption amount of BSA in different nanoparticle treatment groups (n = 5). (**c**) Zeta potential of different nanoparticles. (**d**) Average particle size of different nanoparticles. * Compared with the control group, * *p* < 0.05, ** *p* < 0.01, *** *p* < 0.001, **** *p* < 0.0001; ^#^ was the comparison between groups, ^#^ *p* < 0.05, ^##^ *p* < 0.01, ^###^ *p* < 0.001, ^####^ *p* < 0.0001; ns, *p* > 0.05.

**
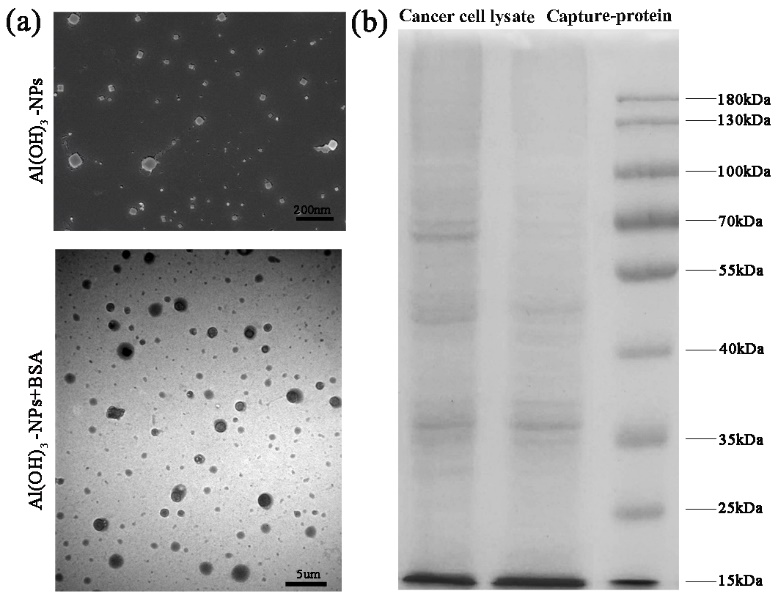
**

Figure S9. (**a**) Changes in morphology and particle size of nano-aluminum hydroxide after protein adsorption. (**b**) In vitro antigen capture validation. SDS-PAGE protein analysis of tumor cell lysate and nano-Al(OH)_3_ capture protein. Samples stained with Coomassie Blue.

**
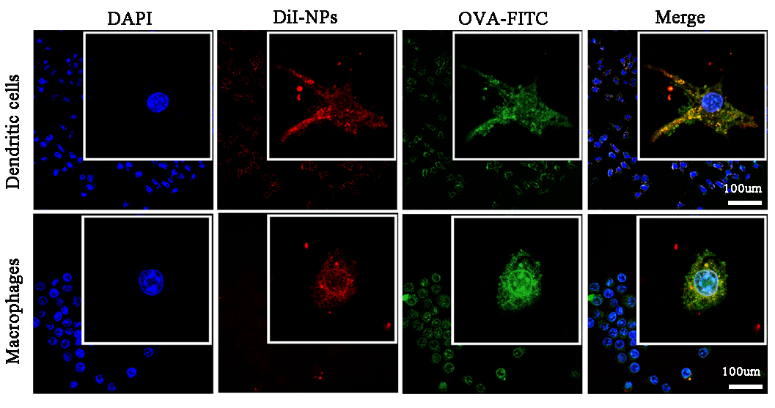
**

Figure 10. Internalization of nanoparticle-captured antigens by DC cells and macrophages. Scale bar 100 μm

**
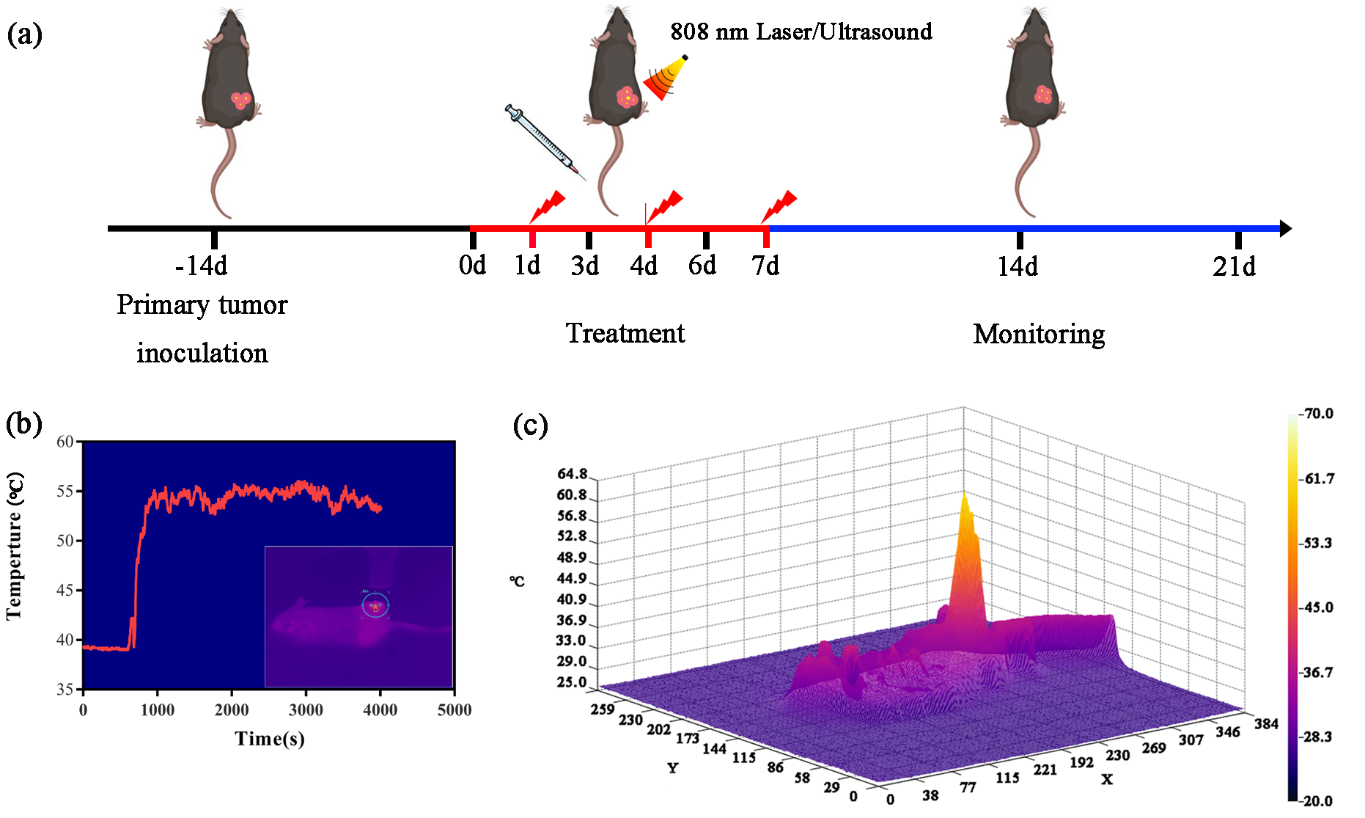
**

Figure S11. In vivo antitumor therapeutic effect. (**a**) PPIAO NPs combined with 808 nm laser and ultrasound treatment inhibited subcutaneous xenografts in female C57BL/6 mice. (**b**) Temperature monitoring of tumor sites irradiated by 808 nm laser after intravenous injection of PPIAO NPs in tumor-bearing C57BL/6 mice. (**c**) Thermal infrared imaging of treated mice. Only the temperature of the tumor irradiation site increased.

**
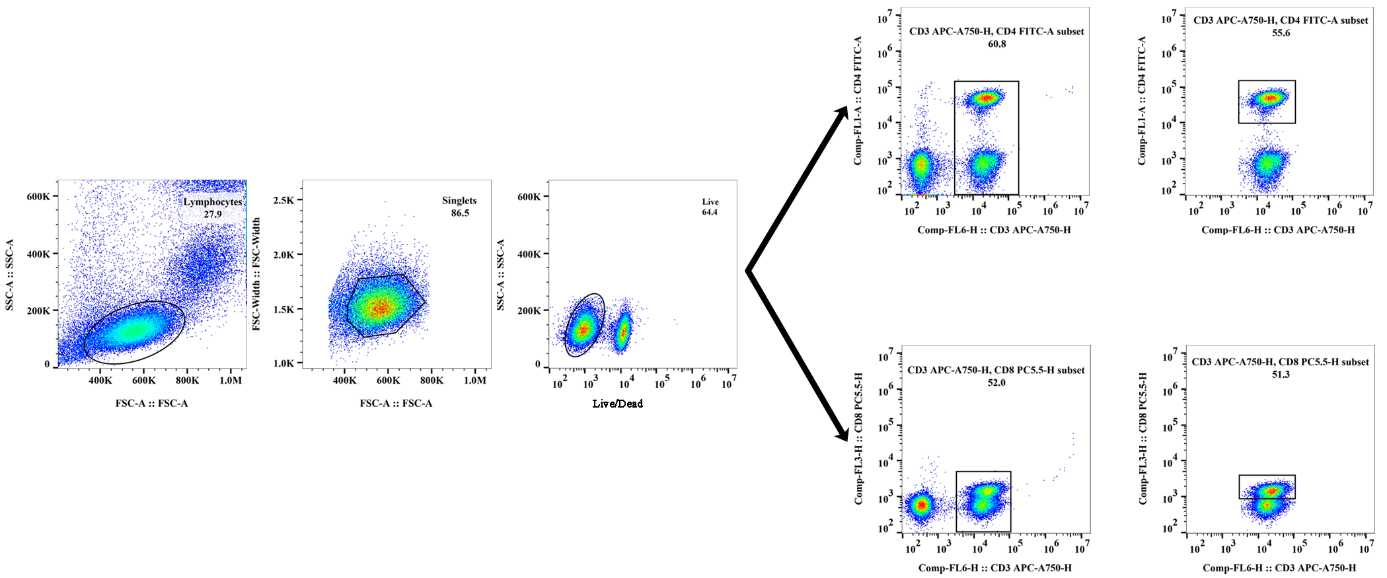
**

Figure S12. Gating strategy for detection of T lymphocytes by FCM.

**
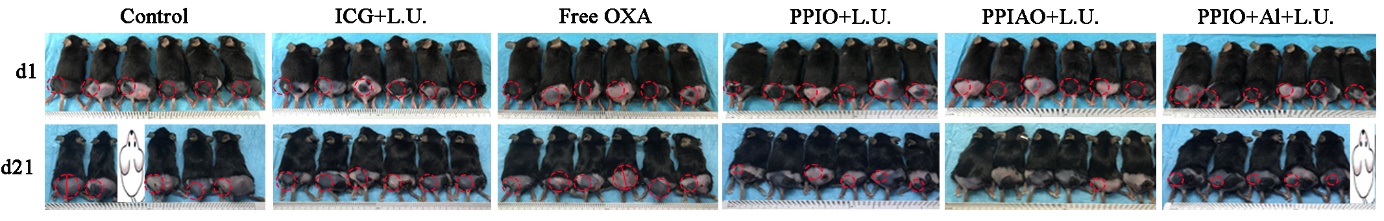
**

Figure S13. Images of primary and metastatic tumors in each goup of female C57BL/6 mice.

**
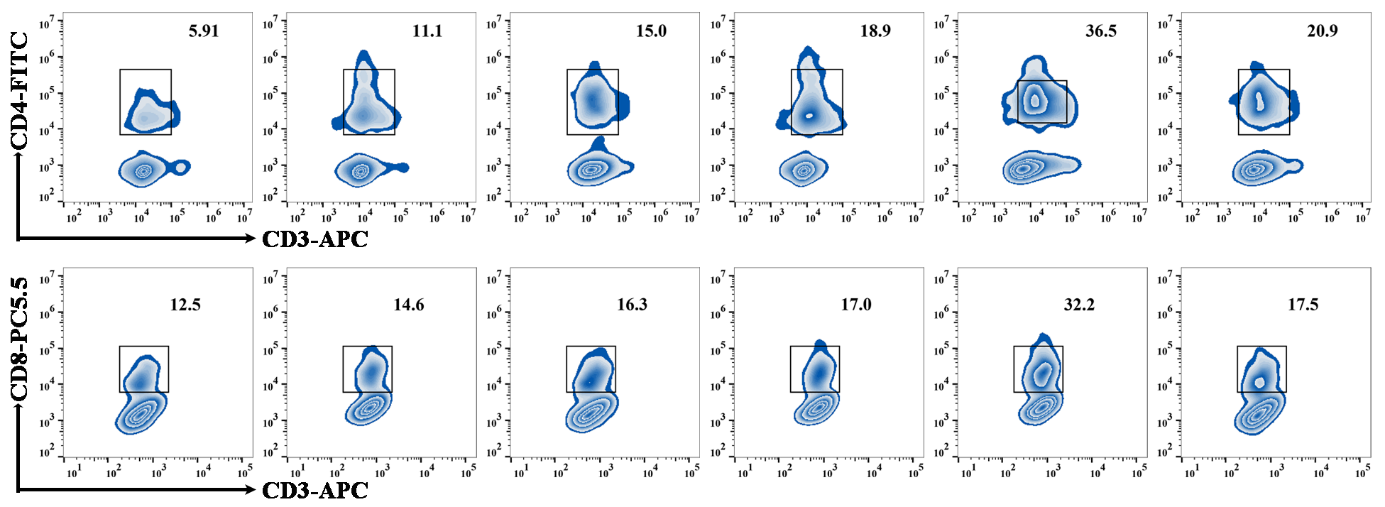
**

Figure S14. FCM detection of CD4^+^ T and CD8^+^ T lymphocytes in the spleen of each group of female C57BL/6 mice.

**
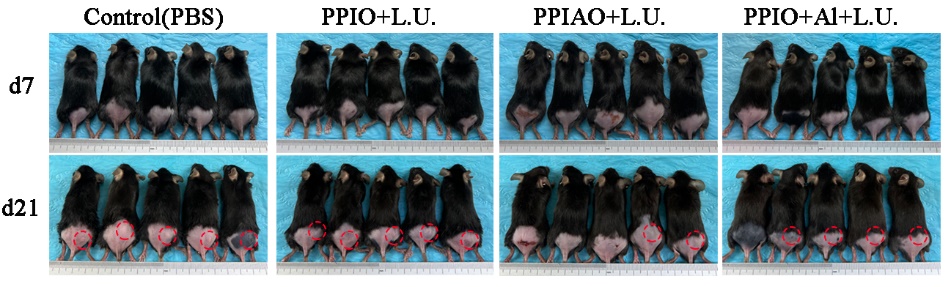
**

Figure S15. Re-challenge of C57BL/6 mice with ID8 cells after ID8 tumor spheroids vaccination.

Table S2. Materials, Antibodies and Chemicals Information

| **Materials, Antibodies and Chemicals(Cat.)** | **SOURCE** |
| --- | --- |
| PEGylated Poly (lactic-co-glycolic acid, lactide: glycolide = 50:50, PLGA 20,000 Da MW, PEG 2000 Da MW) (PLGA-PEG2000) | Ruixi Biotechnology (Xian, China) |
| polyvinyl alcohol (PVA)(341584) | Sigma Aldrich |
| indocyanine green(ICG)(1340009) | Sigma Aldrich |
| Oxaliplatin (OXA)( [HY-17371](https://www.medchemexpress.cn/Oxaliplatin.html)) | MedChemExpress |
| Nano aluminum hydroxide (Al(OH)3) | Ruixi Biotechnology |
| Perfluoropentane (PFP,29℃)(09-6182) | Strem Chemicals |
| APC anti-mouse CD3 Antibody（[E-AB-F1013UE](https://www.elabscience.cn/p-apc_anti_mouse_cd3_antibody_17a2_e_ab_f1013ue-192111.html)） | BioLegend |
| FITC anti-mouse CD4 Antibody（[E-AB-F1097UC](https://www.elabscience.cn/p-fitc_anti_mouse_cd4_antibody_gk1.5_e_ab_f1097uc-192294.html)） | BioLegend |
| [PerCP/Cyanine5.5 Anti-Mouse CD8a Antibody](https://www.elabscience.cn/p-percp_cyanine5.5_anti_mouse_cd8a_antibody_53_6.7_e_ab_f1104uj-192350.html)([E-AB-F1104UJ](https://www.elabscience.cn/p-percp_cyanine5.5_anti_mouse_cd8a_antibody_53_6.7_e_ab_f1104uj-192350.html)) | BioLegend |
| FITC anti-mouse CD11c Antibody([E-AB-F0991UC](https://www.elabscience.cn/p-fitc_anti_mouse_cd11c_antibody_n418_e_ab_f0991uc-192048.html)) | BioLegend |
| APC anti-mouse CD80 Antibody([E-AB-F0992UE](https://www.elabscience.cn/p-apc_anti_mouse_cd80_antibody_16_10a1_e_ab_f0992ue-192060.html)) | BioLegend |
| PE anti-mouse CD86 Antibody([E-AB-F0994UD](https://www.elabscience.cn/p-pe_anti_mouse_cd86_antibody_gl_1_e_ab_f0994ud-192068.html)) | BioLegend |
| CFSE Cell Division Tracker Kit(423801) | BioLegend |
| ER780 anti-mouse/human CD44 Antibody(E-AB-F1100US) | Elabscience |
| PE anti-mouse IFN-γ Antibody([E-AB-F1101UD](https://www.elabscience.cn/p-pe_anti_mouse_ifn_gamma_antibody_xmg1.2_e_ab_f1101ud-192324.html)) | Elabscience |
| [MS Mouse IL-6(Interleukin 6) ELISA Kit](https://www.elabscience.cn/p-ms_mouse_il_6_interleukin_6_elisa_kit-350469.html)(E-MSEL-M0001) | Elabscience |
| [MS Mouse IL-12(Interleukin 12) ELISA Kit](https://www.elabscience.cn/p-ms_mouse_il_12_interleukin_12_elisa_kit-350472.html)(E-MSEL-M0004) | Elabscience |
| [MS Mouse TNF-α(Tumor Necrosis Factor Alpha) ELISA Kit](https://www.elabscience.cn/p-ms_mouse_tnf_alpha_tumor_necrosis_factor_alpha_elisa_kit-350470.html)(E-MSEL-M0002) | Elabscience |
| [MS Mouse IFN-γ(Interferon Gamma) ELISA Kit](https://www.elabscience.cn/p-ms_mouse_ifn_gamma_interferon_gamma_elisa_kit-350475.html)(E-MSEL-M0007) | Elabscience |
| [PCNA Monoclonal Antibody](https://www.elabscience.cn/p-pcna_monoclonal_antibody-77926.html)(E-AB-22001) | Elabscience |
| [TUNEL Assay Kit (HRP-DAB)](https://www.elabscience.cn/p-tunel_assay_kit_hrp_dab_-216233.html)( E-CK-A331) | Elabscience |
| [Anti-Calreticulin antibody (ab92516)](https://www.abcam.cn/calreticulin-antibody-epr3924-er-marker-ab92516.html) | Abcam |
| [Anti-HMGB1 antibody (ab79823)](https://www.abcam.cn/hmgb1-antibody-epr3507-ab79823.html) | Abcam |
| [Anti-CD3 antibody (ab135372)](https://www.abcam.cn/cd3-antibody-sp162-ab135372.html) | Abcam |
| [Anti-CD4 antibody (ab237722)](https://www.abcam.cn/cd4-antibody-cal4-ab237722.html) | Abcam |
| [[Anti-CD8 antibody (ab217344](https://www.abcam.cn/cd8-alpha-antibody-epr21769-ab217344.html))](https://www.abcam.cn/cd8-alpha-antibody-cal66-ab237709.html) | Abcam |
| [Rabbit Anti-Mouse IgG H&L (HRP) (ab6728)](https://www.abcam.cn/rabbit-mouse-igg-hl-hrp-ab6728.html) | Abcam |
| [Goat Anti-Rabbit IgG H&L (Cy5 ®) preadsorbed (ab6564)](https://www.abcam.cn/goat-rabbit-igg-hl-cy5--preadsorbed-ab6564.html) | Abcam |
| Goat Anti-Rabbit IgG H&L (Alexa Fluor® 488) pre adsorbed (ab150081) | Abcam |
| [Bradford Assay Kit (ab102535)](https://www.abcam.cn/bradford-assay-kit-ab102535.html) | Abcam |
| bovine serum albumin(BSA)( A8010) | Solarbio |
| OVA-FITC(SF069) | Solarbio |
| Fetal bovine serum(10099141) | Gibco |
| DMEM/HIGH | Hyclone |
| RPMI1640 | Hyclone |
| PBS .0067M, 500ML | Hyclone |
| 4% paraformaldehyde fixativeSolution(P0099) | Beyotime Biotechnology |
| Antifade Mounting Medium(P0126) | Beyotime Biotechnology |
| ATP Assay Kit(S0026B) | Beyotime Biotechnology |
| Cell Counting Kit-8(CCK8)(C0038) | Beyotime Biotechnology |
| Reactive Oxygen Species Assay Kit (DCFH-DA)(S0033M) | Beyotime Biotechnology |
| LDH Cytotoxicity Assay Kit（C0016） | Beyotime Biotechnology |
| DiI(C1036) | Beyotime Biotechnology |
| DAPI（C1005） | Beyotime Biotechnology |
| Hoechst 33342 Live Cell Stain（C1029） | Beyotime Biotechnology |
| Calcein-AM(CAM) and propidium iodide (PI)（sc-203865，sc-3541） | Santa Cruz Biotechnology |
| [Annexin V-FITC/PI Apoptosis Kit](https://www.elabscience.cn/p-annexin_v_fitc_pi_apoptosis_kit-203045.html)（E-CK-A211） | Elabscience |
| Singlet oxygen sensor green (SOSG)( S36002) | Invitrogen |
| Triton-x100（X100） | Sigma Aldrich |
| Fluoromount(F4680) | Sigma Aldrich |
| ACK Lysis Buffer | Solarbio |
| Fibrinogen | Solarbio |
