# Supplementary material for: Photoacoustic mediated multifunctional tumor antigen trapping nanoparticles inhibit the recurrence and metastasis of ovarian cancer by enhancing tumor immunogenicity
Source: J Nanobiotechnology. 2022 Nov 3;20:468. doi: 10.1186/s12951-022-01682-5 (PMC9632083; doi:10.1186/s12951-022-01682-5)
Supplement: Supplementary file 2 — Additional file 2. In vitro antigen capture validation. SDS-PAGE protein analysis of tumor cell lysate and nano-Al(OH)3 capture protein. Samples stained with Coomassie Blue. [file 12951_2022_1682_MOESM2_ESM.docx]

Additional Information 2

**Photoacoustic mediated multifunctional tumor antigen trapping nanoparticles inhibit the recurrence and metastasis of ovarian cancer by enhancing tumor immunogenicity**

Xiaowen Zhong^1^, Chenyang Li^1^, Guangzong Zhao^1^, Mengmeng Li^1^, Shuning Chen^1^, Yang Cao^2^, Qi Wang^3^, Jiangchuan Sun^1^, Shenyin Zhu^4*^, and Shufang Chang^1*^


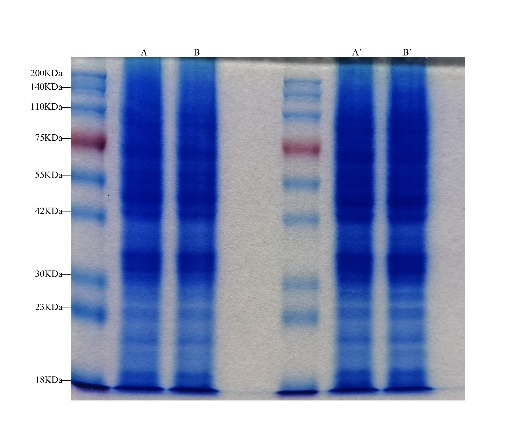

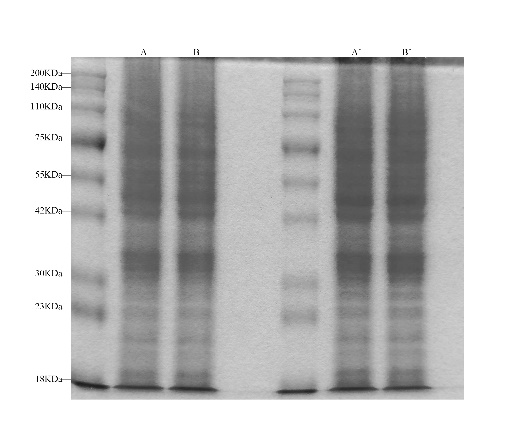


In vitro antigen capture validation. SDS-PAGE protein analysis of tumor cell lysate and nano-Al(OH)_3_ capture protein. Samples stained with Coomassie Blue.
